# Hepatic PRMT1 ameliorates diet-induced hepatic steatosis via induction of PGC1α

**DOI:** 10.7150/thno.63824

**Published:** 2022-02-28

**Authors:** Lu Xu, Zhe Huang, Tak-ho Lo, Jimmy Tsz Hang Lee, Ranyao Yang, Xingqun Yan, Dewei Ye, Aimin Xu, Chi-Ming Wong

**Affiliations:** 1 The State Key Laboratory of Pharmaceutical Biotechnology, The University of Hong Kong, Hong Kong, China.; 2 Department of Medicine, The University of Hong Kong, Hong Kong, China.; 3 Joint Laboratory between Guangdong and Hong Kong on Metabolic Diseases, Guangdong Pharmaceutical University, Guangzhou, China.; 4 Guangdong Research Center of Metabolic Diseases of Integrated Western and Chinese Medicine, Guangdong Pharmaceutical University, Guangzhou, China.; 5 Department of Pharmacology and Pharmacy, Li Ka Shing Faculty of Medicine, The University of Hong Kong, Hong Kong, China.; 6 Department of Health Informatics and Technology, The Hong Kong Polytechnic University, Hong Kong, China.; 7 Hong Kong Polytechnic University Shenzhen Research Institute, Shenzhen, China.

**Keywords:** Non-alcoholic fatty liver disease (NAFLD), Diet-induced hepatic steatosis, PRMT1, PGC-1α, HNF-4α

## Abstract

**Rationale**: Over-nutrition will lead to overexpression of PRMT1 but protein hypomethylation is observed in the liver of obese subjects. The dynamic alteration of the expression and methyltransferase activity of PRMT1 in the progression of fatty liver diseases remains elusive.

**Methods**: We used recombinant adeno-associated virus-mediated gene delivery system to manipulate the hepatic PRMT1 expression level in diet-induced obese mice to investigate the role of PRMT1 in hepatic steatosis. We further utilized a cohort of obese humans with biopsy-proven nonalcoholic fatty liver disease to support our observations in mouse model.

**Results**: We demonstrated that knockdown of PRMT1 promoted steatosis development in liver of high-fat diet (HFD) fed mice. Over-expression of wild-type PRMT1, but not methyltransferase-defective mutant PRMT1^G80R^, could alleviate diet-induced hepatic steatosis. The observation is conserved in the specimens of obese humans with biopsy-proven nonalcoholic fatty liver disease. Mechanistically, methyltransferase activity of PRMT1 was required to induce PGC-1α mRNA expression via recruitment of HNF-4α to the promoter of PGC-1α, and hence attenuated HFD-induced hepatic steatosis by enhancing PGC-1α-mediated fatty acid oxidation.

**Conclusions**: Our results identify that activation of the PRMT1/HNF-4α/PGC-1α signaling is a potential therapeutic strategy for combating non-alcoholic fatty liver disease of obese subjects.

## Introduction

Non-alcoholic fatty liver disease (NAFLD) is a very common condition characterized by high intrahepatic triglyceride content caused by reasons other than excessive alcohol consumption 1. NAFLD is usually asymptomatic in the early stages but will lead to a spectrum of advanced metabolic and liver diseases such as cirrhosis and cancer if left untreated 2. Obesity, type 2 diabetes and dyslipidaemia are well accepted risk factors for NAFLD. In addition, conditions giving rise to dysregulation of hepatic methionine metabolism such as rapid weight loss, starvation, and malnutrition (e.g. folate, B12 and choline deficiency) can also lead to NAFLD 3-5.

Liver is the main organ for the synthesis, utilization and degradation of methyl donors such as S-adenosylmethionine (SAM) and its metabolic intermediates, including S-adenosyl-homocysteine (SAH) and homocysteine 6. Patients with chronic liver disease usually have reduced in SAM biosynthesis 7. SAM is mainly methyl donor for methylation of a large variety of substrates including DNA and protein. SAM concentrations were found significantly depleted in NAFLD patient liver tissues compared to normal liver samples 8. Deficiency in methyl donors would lead to dysregulation of hepatic methionine metabolism and hence induced NAFLD 4, 5. In contrast, dietary supplementation with methyl donors (such as methionine, folate, betaine, and choline) improves glucose homeostasis, attenuates hepatic steatosis, and decelerates the progression of diabetes and NAFLD 9-14.

That is why it was suggested that prolonged consumption of food source without methyl donor supplements leads to hypomethylation of genomic DNA and hence altered expression of hepatic genes involved in lipid metabolism 15 and dietary supplementation with methyl donors reduced fatty liver via modification of fatty acid synthase DNA methylation profile 16. However, indeed, significant number of NAFLD-related genes (such as IGF1, IGFBP2 and PPARG in genomic DNA and MT-ND6 on mitochondrial DNA) underwent hypermethylation with the progression of NAFLD in rodent models as wells as in NAFLD patients 17, 18. DNA methylation could not fully explain the beneficial effects of methyl-donor supplementation in obese subjects on the progression of NAFLD.

In addition to DNA methylation, SAM is also used for protein methylation - an important protein post-translation modification with diverse physiological functions such as signal transduction, mRNA splicing, transcriptional control, DNA repair, and protein translocation 19. The contribution of the protein methylation in NAFLD remains to be explored.

Protein arginine methyltransferases (PRMTs) are the main enzymes that catalyze the transfer of a methyl group from SAM to the guanidino group of arginine residues in target proteins 20. Among the PRMTs, PRMT1 is the predominant member that contributes to ~85% of total arginine methylation 21. Therefore, it is not surprising that PRMT1 regulates many cellular processes such as cell division and proliferation 22. Recent studies reported that hepatic PRMT1 expression level was increased after 16-hr fasting in mice 23 and in obese subjects 23, 24. Interestingly, it was proposed that PRMT1 also regulates the lipogenesis by PGC-1α 24. The evidence was based on PRMT1 siRNA abolished palmitate- or thioredoxin-interacting protein (TXNIP)-induced expression of PGC-1α and lipogenic proteins in rodent liver cell lines 24. Although very little is known about the chronic knockdown or inhibition of hepatic PRMT1 in energy metabolism of obese subjects, targeting of hepatic PRMT1 has been proposed a treatment of NAFLD 24.

It is a paradox that recent studies reported the protective effects of hepatic PRMT1 in alcohol-induced inflammation 25 and hepatocellular carcinoma 26. The potential side effects of targeting hepatic PRMT1 on liver remain largely unknown. In this research, we aimed to discover specifically how PRMT1 regulates hepatic lipid metabolism in obese subjects through comprehensive metabolic phenotyping and deep investigation of molecular mechanisms to enrich our understanding of the role of PRMT1 in hepatic steatosis.

## Materials and methods

### Animal Studies and Tissue Fractionation

8-week-old male C57BL/6N mice were purchased from our Laboratory Animal Unit. Mice were housed in pathogen-free conditions at 22 to 24°C with a 12 hours light-12 hours dark cycle and access to food and water *ad libitum*. All animal experimental procedures were approved by our Committee on the Use of Live Animals for Teaching and Research and were carried out in accordance with the NIH Guide for the Care and Use of Laboratory Animals.

To establish NAFLD mouse model, mice were fed with high-fat diet (HFD, 35% kcal carbohydrates, 20% kcal protein, and 45% kcal fat, D12451, Research Diets Inc., New Brunswick, New Jersey, USA) for 12 weeks starting at the age of 8 weeks, whereas control mice were maintained on a standard chow diet (STC, protein, 18.3%; fat, 10.2%; carbohydrates, 71.5%; D12450B, Research Diets Inc., New Brunswick, New Jersey, USA). For AAV2/8 transduction, 8-week-old male C57BL/6N mice were tail vein injected with 3×10^11^ AAV2/8 vector harboring either PRMT1(WT), PRMT1^G80R^ (Mut) or shPRMT1 with either STC or HFD feeding 27, 28. For adenovirus transduction, 2×10^9^ pfu/mouse adenovirus expressing shRNA against PGC-1α (Vigene Biosciences, Inc., Shandong, China) was injected into the mice with PRMT1 overexpression via the tail vein. The Comprehensive Lab Animal Monitoring System (CLAMS; Oxymax, Columbus, OH, USA) was used to measure food and water intake, volume of oxygen consumption, carbon dioxide, energy expenditure and physical activity. Body composition was measured using ^1^H magnetic resonance spectroscopy (Bruker BioSpin, Billerica, MA, USA) as described previously 29. Isolation of hepatocytes and nonparenchymal cells (NPCs) from liver tissues was performed as described previously 30.

### Recombinant adenovirus, Adeno-associated virus (AAV) Packaging, Concentration and Titration

Previously validated pAV-U6-GFP-PRMT1 small hairpin RNA (shRNA) 31 was purchased from Vigene Biosciences, Inc., Rockville, MD, USA. Full-length cDNAs of luciferase, wild type and mutated PRMT1 were subcloned into the rAAV2-LSP1 vector - downstream of two copies of the hepatic control region of the apolipoprotein E enhancer 29, 32. Each construct was validated by Sanger sequencing. Shuttle vector, together with 2/8 capsids and helper plasmids were then co-transfected in 293T cells with 1 mg/ml polyethyleneimine (PEI, 24314, Polysciences, Inc., PA, USA) for rAAV packaging. rAAV particles were concentrated and purified as described in the standard protocol of AAVanced^TM^ Concentration Reagent (#AAV110A-1, System Biosciences Inc., Palo Alto, CA, USA). The AAV titer was quantified by qPCR as previously described 33. Recombinant adenovirus expressing shRNA targeting PGC-1α or scramble control were purchased from Vigene Biosciences, Inc. Rockville, MD, USA, as previously described 34.

### Measurement of Fatty Acid Oxidation

Fatty acid oxidation for primary cells was measured according to a previous publication 35. 1-^14^C-palmitic acid was purchased from PerkinElmer (56.1 mCi/mmol, #NEC075H, MA, USA). Briefly, freshly harvested liver tissues (100g) were homogenized in chilled STE buffer (250 mM sucrose, 20 mM Tris/HCl pH 7.4, 2mM K-EGTA, 5 mg/ml fatty acid-free BSA) with a 2-mL Dounce homogenizer. Liver homogenate was then incubated with oxidation reaction mixture containing 0.2 μmol cold and 0.8 μCi hot palmitate at 37 °C for 30 minutes. The entire reaction mixture was then transferred to a microcentrifuge tube containing 200 μl of 1 M perchloric acid and a paper disc that has been soaked with 20 μl of 1 M NaOH to trap the ^14^CO_2_. After 1-hour incubation, the paper disc was transferred into a scintillation vial, in which the remaining acid solution was centrifuged and 400 μl of the supernatant was then removed to another new scintillation vial with 4 ml of scintillation fluid. The average counts per minute over 3 minutes were measured with a scintillation counter (LS 6500, Beckman, CA, USA). Data were normalized against total protein contents of samples used in this assay. Fatty acid oxidation for Hepa1-6 cells was measured by FAO assay kit (#E-141, University of Buffalo, NY, USA).

### Histopathologic Analysis

H&E and Oil Red O staining (#O0625, Sigma-Aldrich) were performed on paraffin-embedded and OCT-embedded frozen liver sections, respectively. Detailed procedures of H&E and Oil Red O staining were described previously 36. Representative histopathological images were acquired with a light microscope (Olympus, Tokyo, Japan).

### Liver Lipid Measurements and Enzyme Activity Assays

Liver lipids were extracted as previously described 36. Hepatic triglyceride (TG) and total cholesterol (TC) levels were then measured with commercial kits (#290-63701 for TG and #294-65801 for TC, Wako, Osaka, Japan) according to the manufacturer's instructions. data were normalized against total protein contents of samples used in this assay. Alanine aminotransferase (ALT) and aspartate aminotransferase (AST) levels in the serum were examined using ALT/SGPT LIQUI-UV (#2930-430) and AST/SGOT LIQUI-UV (#2920-430) (Stanbio Laboratory, TX, USA) according to the manufacturer's instructions to evaluate liver function.

### Determination of S-Adenosylmethionine (SAM) Concentration in the Livers of Mice by Mass Spectrometry

The detection method of SAM level in livers were modified from a recent publication 37. In brief, an aliquot of 800 µL of ice cold 80% methanol containing 0.1% formic acid and 10 µmol/L of internal standard (SAM-d3) was added to 20 mg of frozen minced mouse liver tissue. Stainless steel beads (1 mm) were then added to the sample for homogenization with the Precellys Evolution tissue homogenizer (Bertins Instruments, France) at 4500 rpm for 2 min with cool-down intervals. After centrifugation at 15000 g at 4 °C for 10 min, the supernatant was collected and dried with a stream of nitrogen. The dried sample was reconstituted in 800 µL of 5 mM ammonium formate in 80% acetonitrile containing 0.1% formic acid prior Liquid Chromatography/Mass Spectrometry (LC/MS) analysis. Calibration standards spiked with different concentrations of SAM and 10 µmol/L of internal standard (SAM-d3) were prepared in 5 mM ammonium formate in 80% acetonitrile containing 0.1% formic acid for construction of the calibration curve. LC/MS analysis was performed with a Sciex 6500+ QTrap MS connected with a Shimadzu ExionLC LC system. LC separation was performed using a Waters ACQUITY BEH Amide column (1.7µm, 2.1 x 100 mm) with 5 mM ammonium formate containing 0.1% formic acid as solvent A and 5 mM ammonium formate in 80% acetonitrile containing 0.1% formic acid as solvent B. The solvent gradient was 0-9 min: 90%-45% B, 9-10 min: 45%-30% B, 10-11 min: 30%-90% B, 11-14 min: 90% B. The flow rate was set at 0.5 ml/min. The mass spectrometer was operated in positive electrospray ionization and multiple-reaction monitoring mode for detection of SAM (transition: m/z 399→250; declustering potential: 80 V; collision energy: 20 V) and SAM-d3 (transition: m/z 402→250; declustering potential: 80 V; collision energy: 23 V).

### Cell Culture and Luciferase Reporter Assay

Hepa1-6 cells were cultured in DMEM supplemented with 10% FBS, 100 U/mL penicillin and 100 µg/mL streptomycin at 37 °C and 5% CO_2_. The mixture of oleate and palmitate (ratio 2:1, total concentration 0.8 mM) was prepared as described in previous studies 38, 39. Hepa1-6 cells were seeded in 12-well plates and were transfected with 1 mg/ml PEI as indicated for 48 hours. Plasmids overexpressing shRNAs were used to knockdown the expression of either PGC1α or HNF-4α. A scramble shRNA that did not target any gene was synthesized as the negative control. The sequences of the shRNAs are same as reported in previous studies 40-43. Plasmids for expressing shRNAs to knockdown gene expression our *in vitro* studies are either from Vigene Biosciences pre-designed shRNA vectors or inserted the previous validated shRNA into plasmid pLKD-U6-MSC-CMV-Puro. The sequences of shRNAs were summarized in the following Table 1. For luciferase reporter assay, to normalize the transfection efficiency, the pRL-TK (Renilla luciferase) reporter plasmid was used as a transfection control. The luciferase assays were performed by using Dual-Luciferase Reporter Assay System (#E1960, Promega, WI, USA).

### Chromatin Immunoprecipitation (ChIP)-PCR Assay

ChIP assay was performed based on previous reported protocols 44. Hepa1-6 cells were co-transfected with plasmids encoding HNF-4α and WT or Mutant PRMT1. After preclearing the chromatin with protein A-agarose/salmon sperm DNA beads (16-157, Sigma-Aldrich, MO, USA) with rotation for 2 hours at 4 °C, samples were divided equally and incubated with 5 μg of either anti-HNF-4α antibody or non-immune Rabbit IgG (in-house production) with rotation for overnight at 4 °C. DNA was washed and eluted with 30 μl of H_2_O for further analysis by qPCR.

### Western Blot Analysis

Protein from cells or mouse tissues was extracted by RIPA lysis buffer (65 mM Tris-HCl pH 7.5, 150 mM NaCl, 1 mM EDTA, 1% NP-40, 0.5% sodium deoxycholate and 0.1% SDS) as previous described 36. Protein samples were separated on 10% SDS-PAGE gels and transferred to PVDF membranes (IPVH00010, Merck Millipore, CA, USA). The expression of protein was detected by a ChemiDoc MP Imaging System (Bio-Rad, Hercules, CA, USA). Primary antibodies used were: PRMT1 (1:1000, #07-404, Millipore), β-actin (1:1000, #8457, CST), Flag (1:1000, 2368, CST), PGC-1α (1:2000, ab54481, Abcam), HNF-4α (1:1000, ab181604, Abcam), CD11b (1:1000, ab133357, Abcam) and albumin (1:1000, ab207327, Abcam).

### Human Samples

Liver biopsy specimens were collected from 12 morbidly obese patients undergoing bariatric surgery (BMI ≥ 32; 5 men and 7 women). Liver sections with H&E staining were subjected to histological evaluation of steatosis. Simple steatosis was defined by the presence of macrovesicular steatosis affecting at least 5% of hepatocytes without inflammatory foci and evidence of hepatocellular injury in the form of hepatocyte ballooning 45. Individuals with a heavy alcohol-drinking history (≥40 g/day for up to 2 weeks), drug-induced liver disease and hepatitis virus infection were excluded from the study. Clinical parameters of individuals were summarized in Table S1. The human study is approved by the Institutional Review Board of the First Affiliated Hospital of Jinan University, Guangzhou, China (Number: 2019-024). Written informed consent was obtained from participants prior to their inclusion in the study.

### Quantitative Real-time PCR Analysis

Total RNA was extracted with RNAiso Plus (#9109, TaKaRa Bio Inc., Shiga, Japan) as previously described 36. RNA was reverse-transcribed into cDNA with PrimeScript™ RT reagent Kit (#RR037, TaKaRa Bio Inc., Shiga, Japan), and cDNA was then amplified with TB Green Premix Ex Taq (Tli RNase H Plus) Mix (#RR420, TaKaRa Bio Inc., Shiga, Japan), according to the manufacturer's instructions. The RT-qPCR products were analysed using Applied Bio-systems Prism 7000 sequence detection system. The primers used were indicated in the Table 3 below. The mRNA expression levels of the target genes were normalized against the expression of β-actin. Detailed primer sequences were listed below.

### Statistical Analysis

All experiments were performed at least twice, and each experimental group included n ≥ 4 mice. Representative data were shown from two to three independent experiments with similar results. Statistical analysis was performed as described 46. All analyses were performed using SPSS software (version 19.0, IBM), and all data were expressed as means ± standard error of mean (SEM). Statistical differences among two groups were analysed with unpaired 2-tailed Student *t* tests or Mann-Whitney tests for the comparison of variables with or without normal distribution, respectively. Correlation between two groups was assessed by non-parametric Spearman's test. In all statistical comparisons, *P* values were specified as follows: *P < 0.05; **P < 0.01 and ***P < 0.001. NS, not significant.

## Results

### Liver-specific knockdown of PRMT1 exacerbates diet-induced hepatic steatosis

**Figure 1:**
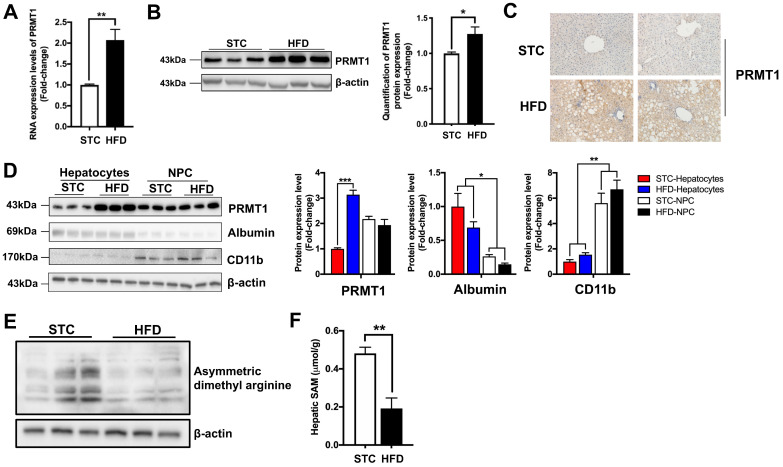
** Long-term HFD feeding markedly induces the expression level of PRMT1 in hepatocytes.** Eight-week-old male C57BL/6N mice were fed with either STC or HFD for 2 months. **A.** Hepatic mRNA expression levels of PRMT1 as determined by qPCR analysis. **B**. Hepatic protein expression level of PRMT1 as determined by Western blotting (left). Each lane is a sample from a different individual. Quantification of hepatic protein expression levels of PRMT1 (right). **C**. Hepatic protein expression level of PRMT1 as determined by Immunohistochemistry (IHC). Representative images of immunohistochemical staining of PRMT1 in liver sections. (200X). **D**. Left panel, Protein expression levels (left panel) of PRMT1, Albumin and CD11b as determined by Western blotting analysis in fractions of hepatocytes or non-parenchymal cell (NPC) isolated from livers of mice fed with either STC or HFD. Right panel, quantification of protein expression levels of PRMT1 (left), Albumin (middle) and CD11b (right). Protein expression levels were normalized to the expression of β-actin. The fraction of Hepatocytes in STC group was set as 1 for fold-change calculation unless mentioned otherwise. Data represent as mean ± SEM; n = 3 per group. **E**. Protein arginine methylation levels in the livers as determined by Western blotting. Each lane is a sample from a different individual. **F**. S-Adenosylmethionine (SAM) concentration in the livers of mice as determined by mass spectrometry. STC group was set as 1 for fold-change calculation unless mentioned otherwise. Data represent as mean ± SEM; n = 4-5 per group; repeated with three independent experiments; *P < 0.05, **P < 0.01, ***P < 0.001.

Consistent with previous studies 23, 24, HFD treatment upregulated hepatic PRMT1 expression at mRNA and protein levels (Figure 1A-C). Tissue fractionation analysis showed that the HFD-mediated induction of PRMT1 expression occurred predominantly in hepatocytes, but not in non-parenchymal cells (NPCs) (Figure 1D), suggesting that hepatocytes are the key contributor to the elevated expression of PRMT1 expression in the livers of HFD-fed mice.

Previous study demonstrated that fasting significantly increase hepatic PRMT1 expression and global asymmetric arginine demethylation in the livers of mice 23. Therefore, we compared the global asymmetric arginine demethylation in the livers of HFD-fed mice with their standard chow (STC)-fed littermates. Interestingly, in contrast to fasting conditions, hepatic proteins were arginine hypomethylated in HFD-fed mice (Figure 1E). Recently study demonstrated that methyl donor SAM level is lowered in obese subjects 8, we also checked the SAM levels in the liver samples obtained from our STC and HFD-fed mice by mass spectrometry. As previously reported 8, we also observed that the SAM level in the liver of HFD-fed mice were only about 50% of STC-fed mice (Figure 1F). Therefore, lower hepatic SAM level limited the methylation capacity of hepatic PRMT1 in the livers of obese subjects 47.

**Figure 2:**
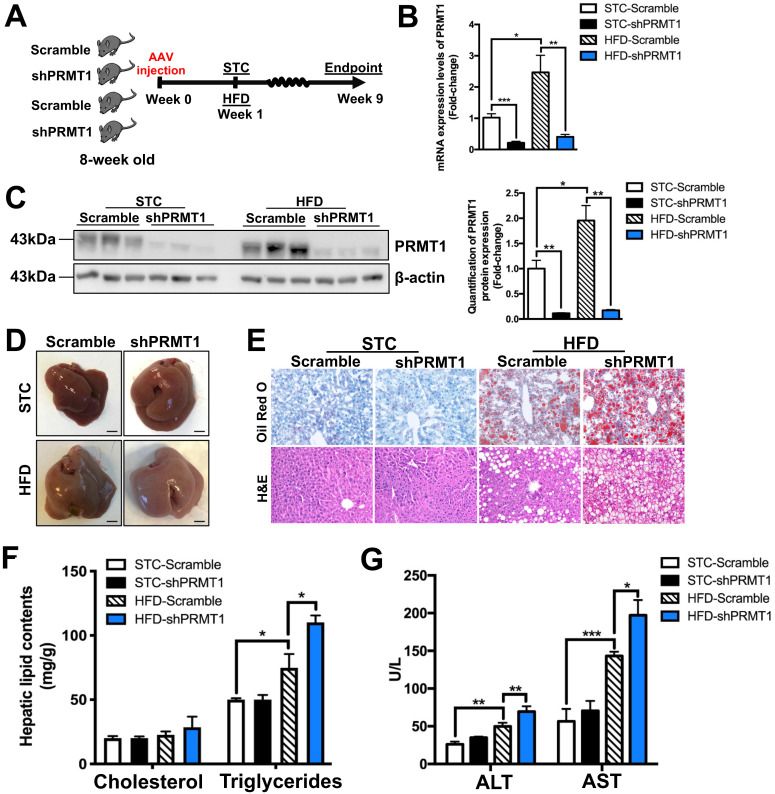
** Knockdown of hepatic PRMT1 exaggerates liver steatosis in mice fed with HFD.** Eight-week-old male C57BL/6N mice were infected with 3×10^11^ copies of AAV encoding U6-PRMT1 shRNA (shPRMT1) or scrambled control (Scramble) shRNA for 9 weeks upon either STC or HFD feeding, respectively. **A.** Schematic illustration of viral treatments. **B**. Hepatic mRNA expression levels of PRMT1 as determined by qPCR analysis. **C**. Hepatic protein expression level of PRMT1 as determined by Western blotting (left). Each lane is a sample from a different individual. Quantification of hepatic protein expression levels of PRMT1 (right). **D.** Representative gross pictures of liver tissue. **E**. Representative images of Oil Red O (upper panel) and H&E (lower panel) staining of liver sections. (200X) **F**. Hepatic cholesterol and triglycerides levels were normalized by total protein contents of liver tissues used for lipid extraction. **G**. Serum levels of alanine transaminase (ALT) and aspartate transaminase (AST). mRNA expression levels of the target genes were normalized to the expression of mouse β-actin. STC-Scramble group was set as 1 for fold-change calculation unless mentioned otherwise. Data represent as mean ± SEM; n = 5-8 per group; repeated with three independent experiments; *P < 0.05, **P < 0.01, ***P < 0.001.

To explore the role of hepatic PRMT1 in energy metabolism in obese condition, we generated rAAV-mediated delivery of shRNA against PRMT1 (shPRMT1) to knockdown hepatic PRMT1 expression in STC and HFD-fed mice (Figure 2A). The knockdown efficiency of hepatic PRMT1 by rAAV-shPRMT1 was confirmed by both quantitative real-time PCR (qPCR, Figure 2B) and Western blotting (Figure 2C) and analyses and shRNA with scramble sequence (shScramble) was used as the control. Almost 90% PRMT1 protein level (Figure 2B) and more than 80% in PRMT1 mRNA expression level (Figure 2C) reduction in the liver of rAAV-shPRMT1 infected mice were observed as compared to rAAV-scramble-infected mice regardless of diet. In addition, no significant differences of the mRNA and protein expression levels of PRMT1 in the epididymal white adipose tissue (eWAT), subcutaneous white adipose tissue (sWAT), soleus muscle (Soleus) and gastrocnemius muscle (Gastroc) in the mice with rAAV-shPRMT1 infection was determined by qPCR (Figure S1A) and observed by immunohistochemical staining (Figure S1B-C).

Although no significant changes in body weight or total body compositions were found between rAAV-shPRMT1 and rAAV-shScramble infected mice under either STC or HFD feeding (Figure S2A-C) and no obvious difference in the gross appearance of livers between the two groups of mice were observed, the liver of HFD-fed mice infected with rAAV-shPRMT1 appeared larger and paler than the control livers infected with rAAV-shScramble (Figure 2D). Consistent with this observation, HFD-induced lipid accumulation within hepatocytes were substantially more abundant in the livers of mice infected with rAAV-shPRMT1 than the controls as determined by Oil Red O staining (Figure 2E; upper panel) and hematoxylin and eosin (H&E) staining (Figure 2E, lower panel). Biochemical tests also showed significantly higher triglyceride content in the liver of rAAV-shPRMT1 infected mice than rAAV-shScramble infected mice (Figure 2F), while serum lipid contents were not affected by knockdown of hepatic PRMT1 (Figure S2D). Importantly, knockdown of hepatic PRMT1 in HFD-fed mice further increased serum levels of alanine transaminase (ALT) and aspartate transaminase (AST), two biomarkers of liver injury, by approximately 39% and 38%, respectively (Figure 2G).

Interestingly, although no significant change in fed and fasting blood glucose levels between rAAV-shPRMT1 and rAAV-shScramble infected mice were observed (Figure S2E-F), hepatic PRMT1 knockdown mice exacerbated HFD-induced glucose intolerance as compared to the controls (Figure S2G), while insulin sensitivity was not significantly affected (Figure S2H). To conclude, these findings collectively suggest that PRMT1 deficiency promotes the development of hepatic steatosis and liver injury in mice.

### Knockdown of PRMT1 reduces hepatic fatty acid oxidation (FAO) capacity in mice

**Figure 3:**
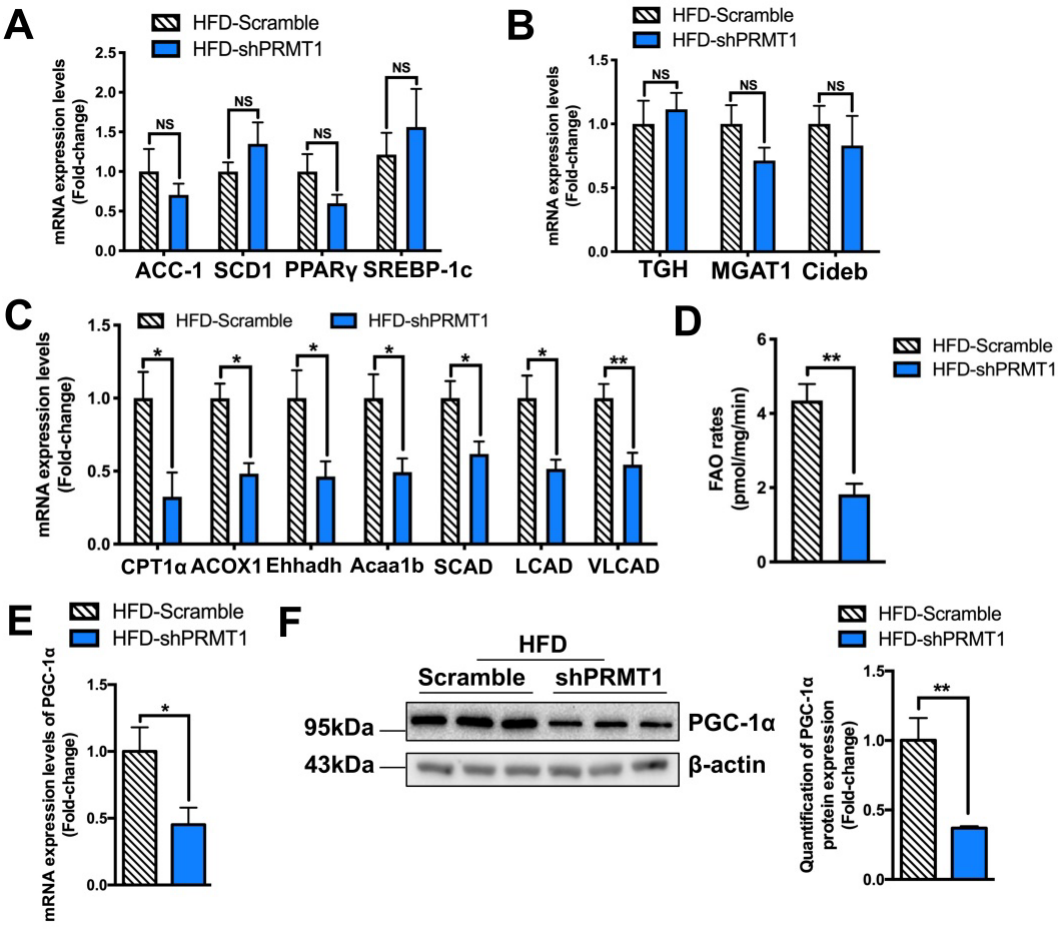
** Down-regulation of hepatic fatty acid *β*-oxidation (FAO) rates is observed in mice with PRMT1 knockdown after long-term HFD feeding.** Eight-week-old male C57BL/6N mice were infected with 3×10^11^ copies of AAV encoding U6-PRMT1 shRNA (shPRMT1) or scrambled control (Scramble) shRNA for 9 weeks upon HFD feeding, respectively. **A.** Hepatic mRNA expression of genes related to lipogenesis (acetyl-CoA carboxylase-1 [ACC-1], stearoyl-CoA desaturase 1 [SCD1] and peroxisome proliferator-activated receptor γ [PPARγ] and sterol regulatory element-binding protein 1 [SREBP-1c]) as determined by qPCR analysis. **B.** Hepatic mRNA expression of genes related to VLDL secretion (Carboxylesterase 3/triacylglycerol hydrolase [TGH], monoacylglycerol acyltransferase 1 [MGAT1], cell death-inducing DFF45-like effector b, [Cideb]) as determined by qPCR analysis. **C.** Hepatic mRNA expression of genes related to FAO (carnitine-dependent transport-1α [CPT1α], acyl‐CoA oxidase 1 [ACOX1], enoyl-CoA hydratase and 3-hydroxyaryl CoA dehydrogenase [Ehhadh], enoyl-CoA hydratase and 3-hydroxyacyl CoA dehydrogenase [Acaa1b], short-chain acyl-CoA dehydrogenases [SCAD], long-chain acyl-CoA dehydrogenases [LCAD] and very long-chain acyl-CoA dehydrogenases [VLCAD]) as determined by qPCR analysis. **D.** Hepatic FAO rates in HFD-Scramble and HFD-shPRMT1 mice were determined by *ex vivo* incubation of freshly harvested mice liver tissues with 1-^14^C-palmitic acid, respectively. Upon incubation, ^14^C-palmitate that does not get oxidized to fatty acyl-CoAs shorter than ∼6 carbons in length will precipitate out of solution upon addition of 1M perchloric acid and left the rest part as incompletely oxidized acid-soluble metabolites (ASMs). In addion, ^14^C-labeled acetyl-CoA will also further enter the tricarboxylic acid (TCA) cycle and be oxidized to ^14^CO_2_. By trapping ^14^CO_2_ using 1M NaOH soaked paper disc. To calculate total FAO rates, we counted the radioactive activity of both ASMs and ^14^CO_2_ with scintillation counter. The average counts per minute of each sample was combined ASMs and ^14^CO_2_ proportion and normalized by total protein contents of liver tissues used in this analysis.** E.** Hepatic mRNA expression levels of PGC-1α as determined by qPCR analysis. mRNA expression levels of the target genes were normalized to the expression of mouse β-actin. HFD-Scramble group was set as 1 for fold-change calculation. **F.** Western blot analysis of Hepatic protein expression of PGC-1α as determined by Western blotting (left). Each lane is a sample from a different individual. Quantification of hepatic protein expression levels of PGC-1α (right). Data represent as mean ± SEM; n = 5-8 per group; repeated with three independent experiments; *P < 0.05, **P < 0.01, NS, not significant.

We next interrogated how down-regulation of hepatic PRMT1 causes steatosis by analysing the expression of hepatic genes involved in lipid metabolism 48. rAAV-shPRMT1 mediated knockdown of hepatic PRMT1 expression had no obvious effects on the expression of key genes involved in lipogenesis (*ACC-1*, *SCD1*, *PPARγ* and *SREBP-1c*; Figure 3A) and VLDL secretion (*TGH*, *MGAT1* and *Cideb*; Figure 3B). Notably, significantly decreased expression of FAO related genes (C*PT1α*, *ACOX1*, *Ehhadh*, *Acaa1b*, *SCAD*, *LCAD* and *VLCAD*; Figure 3C) was observed in the liver of rAAV-shPRMT1 infected HFD-fed mice as compared to the rAAV-shScramble infected HFD-fed mice, suggesting presumably impaired expression of FAO genes in the liver of rAAV-shPRMT1 infected mice.

To further confirm hepatic FAO is impaired in rAAV-shPRMT1 infected mice, their hepatocytes were isolated and examined by *ex vivo* FAO assay 35. Consistent with the lower hepatic FAO-related gene expression, the amount of ^14^CO_2_ produced from oxidation of 1-^14^C-palmitic acid by hepatocytes freshly harvested from rAAV-shPRMT1 infected mice was less than 50% of the scramble controls (Figure 3D), suggesting liver FAO is indeed impaired in hepatic PRMT1 knockdown mice.

Peroxisome proliferator-activated receptor gamma coactivator 1-alpha (PGC-1α) is a key transcriptional coactivator playing critical roles in the maintenance of lipid homeostasis via engagement in numerous metabolic processes, including hepatic FAO 49. Previous studies demonstrated that PRMT1 methylates PGC-1α that contributes to PGC-1α coactivator activity including the induction of genes important for mitochondrial biogenesis 50. PRMT1 siRNA blocked palmitate- or thioredoxin-interacting protein-induced expression of PGC-1α and hence lipogenic proteins in hepatic cell lines 24. We also found a marked reduction in PGC-1α expression in the livers of rAAV-shPRMT1 infected mice at both mRNA (Figure 3E) and protein (Figure 3F) levels as compared to their controls. We also used shRNAs to knockdown PRMT1 expression in palmitic acid (PA) treated murine hepatoma Hepa1-6 cells as prototypes to mimic the hepatocytes in HFD-fed mice, and checked the effects on PGC-1α expression, lipid accumulation and FAO (Figure S3). In brief, the PRMT1 specific shRNAs not only reduced endogenous PRMT1 level (for mRNA by ~ 65% and protein by ~55%), but also reduced endogenous PGC-1α level (mRNA by ~65% and protein by ~50%) in Hepa1-6 cells (Figure S3A-B). In addition, knocking down PRMT1 increased lipid accumulation (Figure S3C-D), and reduced FAO rate of Hepa1-6 cells (Figure S3E). Taken together, our *in vivo* and *in vitro* findings indicate that PRMT1 regulates hepatic lipid metabolism via PGC-1α expression.

### Methyltransferase activity of PRMT1 is required for its protective effects against hepatic steatosis

**Figure 4:**
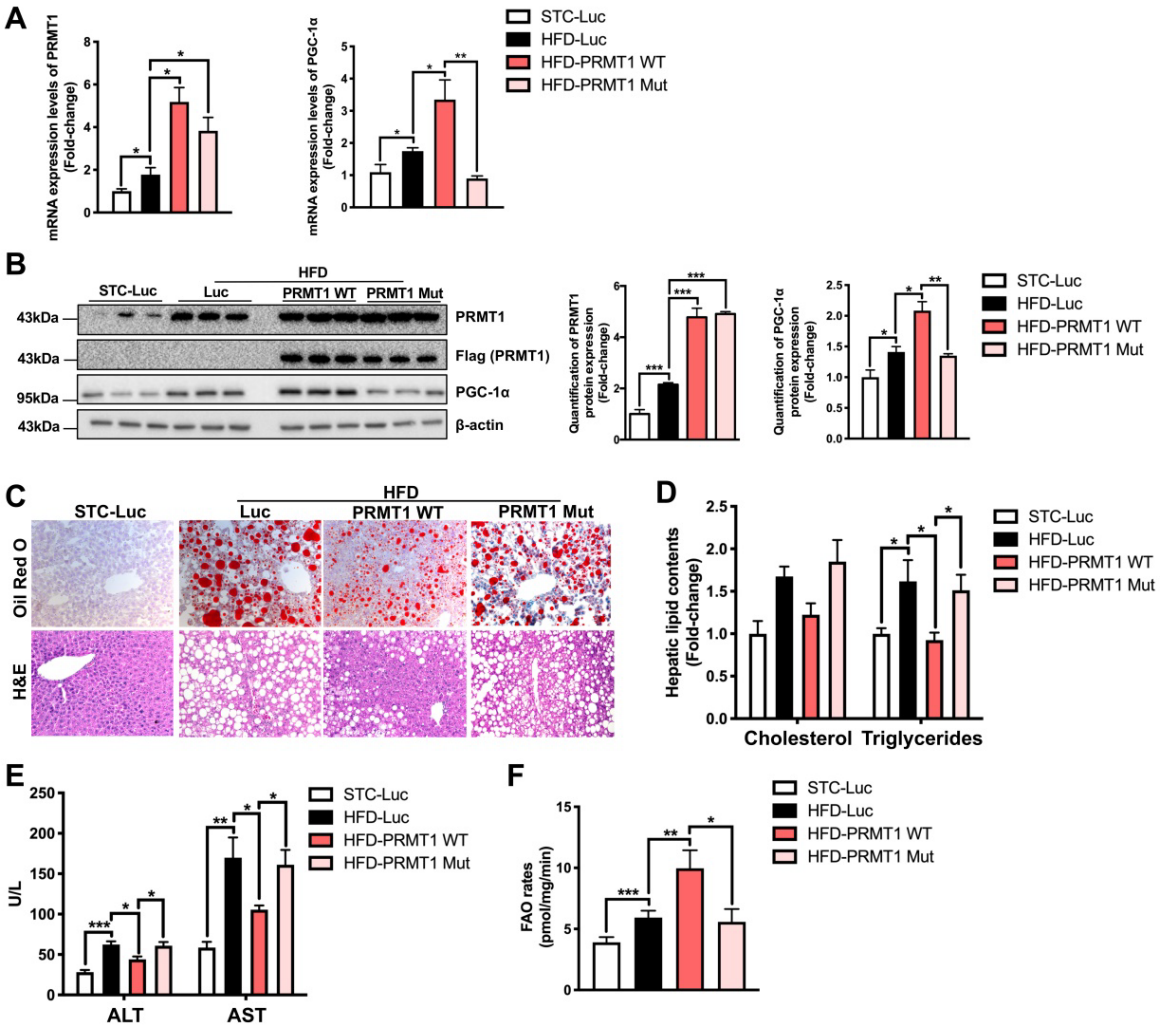
** Methyltransferase inactivation of PRMT1 abolishes its protective role against diet-induced hepatic steatosis.** Eight-week-old male C57BL/6N mice were infected with 3×10^11^ copies of AAV encoding wild-type PRMT1 (PRMT1 WT), methyltransferase activity-deficient PRMT1^G80R^ (PRMT1 Mut) or Luciferase (Luc), for 12 weeks upon HFD feeding. **A**. Hepatic mRNA expression levels of PRMT1 (left) and PGC-1α (right) as determined by qPCR analysis. **B**. Protein expression of PRMT1 and PGC-1α in the liver tissue of these mice as determined by Western blotting (left). Each lane is a sample from a different individual. Quantification of hepatic protein expression levels of PRMT1 (middle) and PGC-1α (right). **C**. Representative images of Oil Red O (upper panel) and H&E (lower panel) staining of liver sections. (200X) **D.** Hepatic cholesterol and triglycerides levels were normalized by total protein contents of liver tissues used for lipid extraction. **E.** Serum ALT and AST levels. **F.** Hepatic FAO rates were determined by *in vitro* incubation of freshly harvested mice liver tissues with 1-^14^C-palmitic acid as shown in Figure 3B. The average counts per minute of each sample was combined ASMs and ^14^CO_2_ proportion and normalized by total protein contents of liver tissues used in this analysis. mRNA expression levels of the target genes were normalized to the expression of mouse β-actin. STC-Luc group was set as 1 for fold-change calculation. Data represent as mean ± SEM; n = 5-8 per group; repeated with three independent experiments; *P < 0.05, **P < 0.01, ***P < 0.001.

To validate the findings above, we further interrogated whether liver-specific overexpression of PRMT1 protects against diet-induced liver steatosis. rAAV expressing wild-type PRMT1 (PRMT1-WT) under liver-specific ApoE enhancer was used to overexpress PRMT1 in livers. Given that a single amino acid mutation G80R introduced in the conserved methyl donor SAM binding domain of PRMT1 has been shown to impair its methyltransferase activity 51, 52. We also generated rAAV for gene delivery of methyltransferase-defective form of PRMT1 by introducing G80R mutation (PRMT1-Mut) to test whether methyltransferase activity is required for the protective role of PRMT1 in NAFLD. In addition, rAAV-ApoE-Luciferase (Luc) was used as another negative control in our experiments. Successful over-expression of PRMT1 in livers of mice infected with either PRMT1-WT or PRMT1-Mut was confirmed at both mRNA and protein levels by and qPCR (Figure 4A) Western blotting (Figure 4B) respectively. We also used immunohistochemistry and tissue fractionation analysis showed that the PRMT1 was mainly overexpressed in hepatocytes (Figure S4). rAAVs of Luc, PRMT1-WT or PRMT1-Mut showed no significant effects on body weight and compositions upon HFD feeding (Figure S5A-C).

Since the expression of PGC-1α, the master regulator of FAO 49, was downregulated in the liver of PRMT1 knockdown mice, we further compared the expression level of PGC-1α in the liver of mice overexpressing PRMT-WT or PRMT-Mut. Interestingly, the expression of PGC-1α at both mRNA (Figure 4A right panel) and protein (Figure 4B left and right panel) and levels was significantly induced by overexpression of PRMT1-WT, but not for PRMT1-Mut (Figure 4A-B).

Intriguingly, consistent with the knockdown findings mentioned above, overexpression of PRMT1-WT in liver attenuated the development of HFD-induced steatosis as evident by the lower hepatic lipid accumulation in rAAV-PRMT1-WT infected mice as compared to the rAAV-Luc group (Figure 4C) as well as lower hepatic triglyceride content (Figure 4D). Overexpression of PRMT1-WT also ameliorated liver injury in HFD-fed mice as shown by lower serum ALT and AST in mice receiving rAAV-PRMT1-WT then the rAAV-Luc infected mice (Figure 4E). In addition, *ex vivo* FAO assay indicated higher hepatic FAO rate in rAAV-PRMT1-WT infected mice than rAAV- Luc infected mice (Figure 4F). Interestingly, such improvements were not observed in mice infected with rAAV overexpressing the methyltransferase activity-deficient mutant PRMT1-Mut (Figure 4C-F). Taken together, our results indicate that methyltransferase activity of PRMT1 is indispensable for its protective role against diet-induced hepatic steatosis in mice. These findings also suggest that fully active PRMT1 enhances hepatic FAO possibly via the induction of PGC-1α expression.

### PRMT1 enhances fatty acid oxidation and attenuates hepatic steatosis via PGC-1α

**Figure 5:**
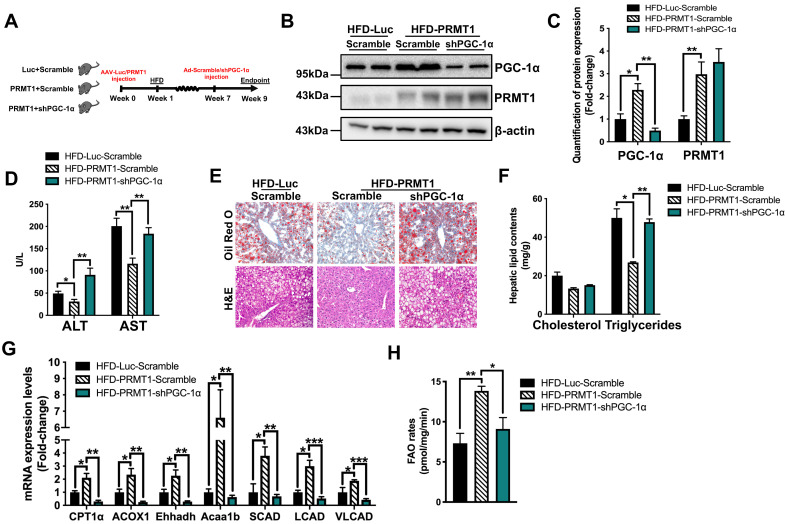
** PGC-1α is required for PRMT1-mediated alleviation of diet-induced hepatic steatosis.** Eight-week-old male C57BL/6N mice were infected with 3×10^11^ copies of AAV encoding ApoE-PRMT1 (PRMT1) for over-expression of PRMT1 or ApoE-Luciferase control (Luc) for 7 weeks upon HFD feeding. Adenovirus encoding PGC-1α shRNA (shPGC-1α) or scrambled control (Scramble) were subsequently given by tail vein injection (2×10^9^ p.f.u./mouse) at 7 weeks post AAV injection and mice were sacrificed 2 weeks post-adenoviral infection. **A**. Schematic illustration of viral treatments. **B.** Hepatic protein expression of PGC-1α and PRMT1 as determined by Western blotting. Each lane is a sample from a different individual. **C**. Quantification of hepatic protein expression of PGC-1α and PRMT1. **D**. Serum levels of ALT and AST. **E**. Representative images of Oil Red O (upper panel) and H&E (lower panel) staining of liver sections. (200X) **F**. Hepatic cholesterol and triglycerides levels were normalized by total protein contents of liver tissues used for lipid extraction. **G.** qPCR analysis of mRNA expression levels of hepatic FAO related genes. **H.** Hepatic FAO rates were determined by *ex vivo* incubation of freshly harvested mice liver tissues with 1-^14^C-palmitic acid as shown in Figure 3B. The average counts per minute of each sample was combined ASMs and ^14^CO_2_ proportion and normalized by total protein contents of liver tissues used in this analysis. mRNA expression levels of the target genes were normalized to the expression of mouse β-actin. HFD-Luc-Scramble group was set as 1 for fold-change calculation. Data represent as mean ± SEM; n = 5-8 per group; repeated with three independent experiments; *P < 0.05, **P < 0.01, ***P < 0.001.

We next investigated whether the stimulatory effects of PRMT1 on hepatic FAO is dependent on PGC-1α. To this end, we treated rAAV-PRMT1-WT HFD-fed mice with recombinant adenovirus expressing shRNA targeting PGC-1α or scramble control (Figure 5A). The hepatic protein expression of PGC-1α was repressed by approximately 80% in mice treated with shRNA against PGC-1α as compared to scramble control, while the expression of hepatic PRMT1 was not affected (Figure 5B-C). Overexpression of PRMT1-WT consistently showed protective effects against diet-induced hepatic steatosis (Figure 5D-H). Notably, these protective effects induced by PRMT1 overexpression were largely abolished in PGC-1α knockdown mice, including lowering of serum levels of liver injury markers (Figure 5D), reduction of lipid accumulation in the liver (Figure 5E-F) and induction of hepatic FAO (Figure 5G-H). We also confirmed the results with *in vitro* assays by overexpressing PRMT1 in Hepa1-6 cells(Figure S6-7). Transfection of PRMT1 overexpressing plasmid into Hepa1-6 cells not only increased the PRMT1 level (mRNA by ~8 folds and protein by ~3.3 folds), but also increase PGC-1α level (mRNA by ~ 3 folds and protein by ~2.1 folds; Figure S6A-B) Overexpression of PRMT1 could lower PA-induced lipid accumulation (Figure S6C-D) and increased FAO rates (Figure S6E). However, such effects of PRMT1 overexpression on elevation of FAO rate and reduction of PA-induced lipid accumulation was largely abrogated by knockdown of PGC-1α expression in Hepa1-6 cells (Figure S7A-E). As shown in Figure S7A-B, PGC-1α specific shRNAs could knockdown endogenous PGC-1α level in PRMT1 overexpressing Hepa1-6 cells (mRNA by ~80% and protein by ~65%; Figure S7A-B), abolished the alleviation of lipid accumulation (Figure S7C-D) and enhancement of FAO rate (Figure S7E) mediated by PRMT1 overexpression. Taken together, PGC-1α is required for PRMT1-mediated protective effects against hepatic steatosis.

### PRMT1 induces the expression of PGC-1α via HNF-4α

**Figure 6:**
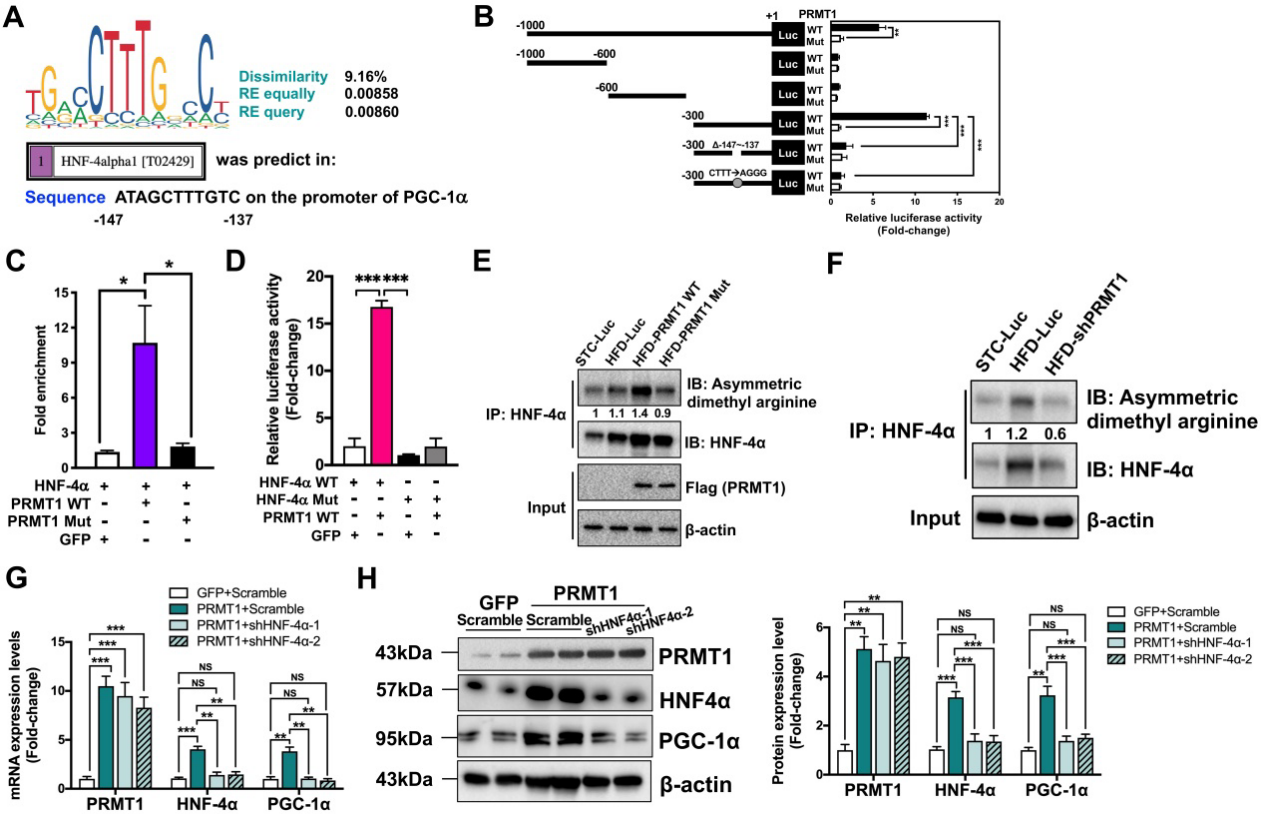
** The methyltransferase activity of PRMT1 regulates the transcriptional activity of PGC-1α through HNF-4α. A**. DNA motif sequence logo of mouse HNF-4α was generated by JASPAR (2018). The dissimilarity, random expectation (RE) equally and query between query sequence of PGC-1α and predicted transcription factor binding site sequence of HNF-4α were calculated using PROMO (ver. 8.3). **B**. Luciferase reporter assay on PGC-1α promoter in response to over-expression of WT or Mutant PRMT1 by truncation analysis. Hepa1-6 cells were co-transfected with pAM2AA-ApoE-PRMT1 WT or Mutant (Mut) plasmids with different lengths or mutant (deletion of -146 to -137 region) of pGL3-PGC-1α promoter-Luciferase plasmids for 48 hours. Cell lysates were used for luciferase assay. Total protein content of each sample was used for normalization. Treatment with co-transfection with PRMT1 Mut and promoter region -1000/+1 of PGC-1α was set as 1 for fold-change calculation. **C**. Fold enrichment of occupancy of HNF-4α on the promoter region of PGC-1α in response to co-expression of HNF-4α with GFP/PRMT1 WT/PRMT1 Mut in Hepa1-6 cells as detected by ChIP assay with rabbit anti-HNF-4α polyclonal antibody or non-immune rabbit IgG as control. The precipitated chromatin was analysed by qPCR using primers (shown in Table S2) spanning to the PGC-1α proximal promoters. **D.** Luciferase reporter assay on PGC-1α promoter in response to over-expression of WT or non-methylatable Mutant (Mut) HNF-4α plasmids. Hepa1-6 cells were co-transfected with pAM2AA-ApoE-PRMT1 WT/GFP with either pAM2AA-ApoE-HNF-4α WT or Mut plasmids with pGL3-PGC-1α promoter (-300 to +1)-Luciferase plasmids for 48 hours. Cell lysates were used for luciferase assay. Total protein content of each sample was used for normalization. Treatment with co-transfection with HNF-4α WT, GFP and promoter region -300/+1 of PGC-1α was set as 1 for fold-change calculation. mRNA expression levels of the target genes were normalized to the expression of mouse β-actin. Treatment with co-transfection with GFP and HNF-4α was set as 1 for fold-change calculation. mRNA expression levels of the target genes were normalized to the expression of mouse β-actin. Data represent as mean ± SEM; n = 5-8 per group; repeated with three independent experiments; *P < 0.05, **P < 0.01, ***P < 0.001. **E.** Methylation levels of HNF-4α in liver lysates harvested from STC-Luc, HFD-Luc, HFD-PRMT1 WT and HFD-PRMT1 Mut mice were analysed, and **F**. Methylation levels of HNF-4α in liver lysates harvested from STC-Luc, HFD-Luc, HFD-shPRMT1 mice were analysed by immunoblotting using anti-dimethyl arginine antibody. Relative amounts of methylated HNF-4α over total HNF-4α was determined by densitometry and indicated below the blots. HNF-4α is required for PRMT1-mediated induction of PGC-1α expression on Hepa1-6 cells. Hepa1-6 was transfected with either GFP or PRMT1 over-expressing plasmids for 24 hours, followed by transfection with shScramble (Scramble) or shHNF-4α-1 or shHNF-4α-2 plasmids for another 72 hours. **G**. mRNA expression levels of PRMT1, HNF4α and PGC-1α as determined by qPCR analysis. **H**. Protein expression of PRMT1, HNF-4α and PGC-1α as determined by Western blotting (left). Quantification of protein expression levels of PRMT1, HNF4α and PGC-1α (from left to right). mRNA expression levels of the target genes were normalized to the expression of mouse β-actin. Control group was set as 1 for fold-change calculation. Data represent as mean ± SEM; n = 4 per group; repeated with three independent experiments; *P < 0.05, **P < 0.01, ***P < 0.001, NS, not significant.

Next, we explored how PRMT1 induces PGC-1α expression. Hepatocyte nuclear factor 4α (HNF-4α), a nuclear receptor expressed predominantly in liver, plays a critical role in transcriptional regulation of genes involved in liver development and energy metabolism 53. Knockdown hepatic HNF-4α resulted in development of fatty liver in mice 41, and upregulating HNF-4α expression suppressed hepatic lipid accumulation rats fed with HFD 54. Furthermore, HNF-4α loss-of-function mutations in human are associated with maturity-onset diabetes of the young and lipid disorders 55, 56. Notably, HNF-4α has been identified as a substrate of PRMT1 57, 58. PRMT1 methylates HNF-4α DNA binding domain to enhance its binding affinity, and PRMT1 is further recruited by HNF-4α to the promoter/enhancer regions of target genes for transcriptional activation by methylation of histone H4 arginine 3 57, 58. A potential HNF-4α binding site was identified on the promoter region of PGC-1α gene (between -147 to -137 from the transcription start site; Figure 6A). Therefore, we hypothesized that the expression of PGC-1α is mediated by PRMT1 through HNF-4α.

In order to test this hypothesis, dual luciferase reporter assay with truncated mouse PGC-1α promoter constructs were used to validate whether the putative HNF-4α binding site is required for the induction of PGC-1α expression in a PRMT1 methyltransferase-activity dependent manner (Figure 6B left panel). Each construct was transiently co-transfected into murine hepatoma Hepa1-6 cells, with plasmid expressing either PRMT1-WT or PRMT1-Mut. Transcriptional activity of the constructs was quantified by measuring the luciferase activity. To this end, the luciferase activities of the constructs harboring the predicted HNF-4α binding site (-147/-137) were induced by overexpression of PRMT1-WT, but not PRMT1-Mut (Figure 6B right panel). Mutation of the putative HNF-4α binding site from ATAGCTTTGTC to ATAGAGGGGTC also could abolish the induction by overexpression of PRMT1-WT. Therefore, the predicted HNF-4α binding site on PGC-1α promoter and methyltransferase activity of PRMT1 were required for the induction of PGC-1α expression.

To further confirm whether HNF-4α is indeed recruited to the predicted HNF-4α binding site on PGC-1α promoter, chromatin immunoprecipitation-quantitative PCR (ChIP-qPCR) assay was performed with rabbit anti-HNF-4α polyclonal antibody (Figure 6C). In addition to transfection of plasmid encoding HNF-4α, Hepa1-6 cells were also co-transfected with plasmid overexpressing green fluorescent protein (GFP), PRMT1-WT or PRMT1-Mut. Based on the PGC-1α promoter DNA fragments pulled down by HNF-4α antibody and quantified by qPCR (Figure 6C), there was a strong interaction between HNF-4α and the promoter of PGC-1α upon overexpression of PRMT1-WT, but not GFP or PRMT1-Mut (Figure 6C).

To validate the importance of the methylation site of HNF-4α in PGC-1α expression, we constructed the non-methylatable HNF-4α mutant R91W (HNF-4α Mut) 57, 58 and repeated the dual luciferase reporter assay in with pGL3-PGC-1α promoter (-300 to +1)-Luciferase. As shown in Figure 6D, the luciferase activities could only be induced by overexpressing the wild-type HNF-4α, but not the HNF-4α^R91W^ (HNF-4α Mut). To assess the methylation level of HNF-4α in the livers of STC and HFD-fed mice, endogenous hepatic HNF-4α was immune precipitated by HNF-4α followed by Western blotting using anti-dimethyl arginine antibody. The representative result in Figure 6E showed hepatic HNF-4α methylation decreased by HFD treatment in mice. Intriguingly, overexpression of the wild-type PRMT1, but not the catalytically-inactive PRMT1^G80R^ (PRMT1 Mut), can increase the hepatic HNF-4α methylation. In addition, we knocked down the endogenous hepatic PRMT1 to further confirm the PRMT1 is the key methyltransferase that methylates HNF-4α (Figure 6F). Consistent with the *in vivo* data above, shRNAs-mediated knockdown of endogenous HNF-4α expression abolished the effects of PRMT1-induced of PGC-1α expression in Hepa1-6 cells. As shown in Figure 6G-H, HNF-4α specific shRNAs could reduce the both endogenous HNF-4α (mRNA by ~80% and protein by ~60%) PGC-1α (mRNA by ~75% and protein by ~55%) expression level, but did not affect the expression level of PRMT1. In summary, methylation of HNF-4α by PRMT1 is required for the recruitment of HNF-4α to PGC-1α promoter and hence induction of PGC-1α expression.

### Dysregulated induction of PRMT1 and PGC-1α in liver is closely associated with hepatic steatosis in obese patients

**Figure 7:**
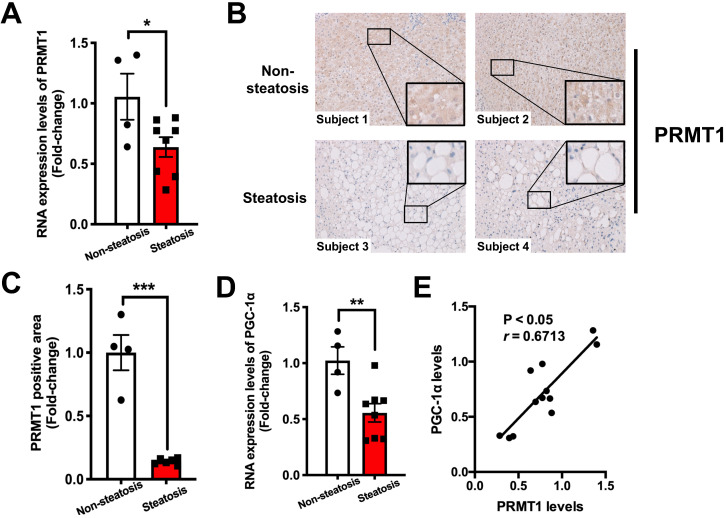
** Hepatic expression of PRMT1 is downregulated in obese patients with hepatic steatosis when compared to those with normal liver morphology, and is positively associated with hepatic PGC-1α expression level. A.** Hepatic IHC staining of PRMT1 in liver sections of these study subjects. (200X) **B.** Quantification of PRMT1 protein expression levels in liver sections of these study subjects based on IHC staining results by using ImageJ. **C.** mRNA expression levels of PRMT1 and **D.** mRNA expression levels of PGC-1α in liver of these study subjects as determined by qPCR analysis. **E.** Correlation between PRMT1 and PGC-1α levels in liver of these study subjects. mRNA expression levels of the target genes were normalized to the expression of human β-actin. Obese subjects with healthy liver were set as 1 for fold-change calculation. Data represent as mean ± SEM; n = 4-8 per group. Correlation was assessed by non-parametric Spearman's test. *P < 0.05, **P < 0.01, ***P < 0.001.

To evaluate the clinical relevance of our findings in animal studies, liver biopsies collected from 12 morbidly obese (BMI ≥ 32) subjects were subjected to histological evaluation of the degree of steatosis using non-alcoholic steatohepatitis clinical research network (NASH CRN) scoring system 59. We checked the expression of PRMT1 and PGC-1α in liver biopsies with severe steatosis (n = 8) and those with absence of hepatic steatosis (n = 4) by qPCR and immunostaining analysis. Immunostaining analysis demonstrated that hepatic expression of PRMT1 protein was lower in the ones with steatosis than those with lower liver fat content (Figure 7A and B). Consistent with our findings with HFD-fed mice, hepatic mRNA levels of both PRMT1 and PGC-1α were lower in obese patients with steatosis as compared to the obese subjects with low liver fat content (Figure 7C and D). Furthermore, the expression level of PRMT1 was positively correlated with PGC-1α in the liver (r = 0.6713, *P* < 0.05; Figure 7E). These data collectively suggest that impairing hepatic PRMT1 and PGC-1α induction correlates hepatic steatosis in obese subjects.

## Discussion

This study was initiated by two apparently paradoxical observations. On one hand, based on *in vitro* assays and rodent studies, knockdown hepatic PRMT1 expression was suggested for the treatment of NAFLD 24. On the other hand, the key methyl donor SAM was dramatically depleted in obese NAFLD subjects 8 and methyl-donor supplementation halts the progression of NAFLD 6, 14, 60, 61. The role of this predominant protein arginine methyltransferases PRMT1 in hepatic steatosis remains poorly defined. Our findings suggest that the increase in PRMT1 expression in liver in obesity serves as a compensatory mechanism for the decrease in SAM to alleviate HFD-induced hepatic steatosis and liver damage by promoting fatty acid oxidation.

In this study, we firstly investigated the expression level of PRMT1 and protein methylation in the liver of obese mice. Then, we provided several lines of *in vivo* evidence demonstrating the importance of the PRMT1 in alleviating hepatic steatosis by using rAAV mediated tissue-specific gene delivery, including chronic knockdown of hepatic PRMT1 exacerbated hepatic steatosis and liver injury in HFD-fed mice, and chronic overexpression of PRMT1, but not the methyltransferase defective mutant PRMT1^G80R^ in the liver alleviated HFD-induced hepatic steatosis.

PGC-1α is a pivotal transcriptional coactivator of energy metabolism 62, 63, especially in promoting hepatic lipid catabolism 49, 64, 65. Previous study showed that over-expression of PGC-1α in the liver in rats resulted in increased FAO and reduced lipid accumulation in hepatocytes 49. Whereas, mice with liver-specific knockdown of PGC-1α or PGC-1α null mice diminished hepatic FAO capacity and enhanced fasting-induced hepatic steatosis phenotypes 65, 66. Consistent with these publications, we demonstrated that PGC-1α is an obligatory downstream effector of PRMT1 that mediate hepatic FAO capacity and subsequent protective effects of overexpressing PRMT1 against HFD-induced steatosis. Firstly, HFD-induced expression of hepatic PGC-1α was suppressed in mice with shRNA-mediated knockdown of PRMT1. Secondly, the methyltransferase activity of PRMT1 was required to restore the expression of PGC-1α in the liver. Thirdly, the beneficial effects of PRMT1 overexpression in reduction of hepatic lipid accumulation in obese mice and induction of hepatic FAO gene expression and FAO capacity were largely abrogated by shRNA-mediated knockdown of PGC-1α expression in the liver.

Despite extensive studies focused on the transcription activity of PGC-1α via methylation by PRMT1 on its activity to induce the expression of its target genes 50, the mechanism of how PRMT1 induces of PGC-1α expression in the liver are not well defined. Indeed, HNF-4α has been found as a substrate of PRMT1 57, 58. Our present study provided both *in vitro* and *in vivo* evidence firstly demonstrating that the methyltransferase activity of PRMT1 is required to induce PGC-1α mRNA expression via recruitment of HNF-4α to the promoter of PGC-1α. Therefore, we conclude hepatic PRMT1 ameliorate diet-induced hepatic steatosis via induction of PGC-1α expression by methylation of transcription factor HNF-4α.

Previous study demonstrated that PRMT1 enhances hepatic lipogenesis by increasing lipogenic gene expression via the TXNIP/PRMT1/PGC-1α pathway 24. Their evidence was mainly based on *in-vitro* experimental data with knockdown of PRMT1 in AML12 and H4IIE cells by siRNAs. In contrast, *in vivo* approaches with rAAV-mediated chronic gene delivery were employed in our study. We demonstrated that overexpression of hepatic PRMT1 in HFD-fed mice for 9-week alleviated hepatic steatosis by enhancing FAO in their livers. The opposite observations shall be due to substantial differences between *in vitro* and *in vivo* experiments.

Given that the amino acid sequences of human and mouse PRMT1 proteins share 99% identity and the expression level of PRMT1 and PGC-1α negatively correlated with liver fat content are conserved between human and mouse, we expect the underlying regulatory mechanism is similar. Interestingly, recent studies reported that PRMT1 could protect alcohol-induced liver injury and suppress alcohol‐induced hepatocellular carcinoma formation in mice 25, 26. We also observed that the expression level of the liver injury markers ALT and AST were higher in obese humans with low hepatic PRMT1 expression (Table S1, *P* = 0.07 for ALT and *P* = 0.08 for AST). As our sample size is small to draw concrete conclusion at this point, it will be interesting to explore whether overexpression of hepatic PRMT1 can also protect against NAFLD-induced liver injury in other large cohort studies.

In summary, we uncover the protective role of hepatic PRMT1 against liver steatosis in obese subjects by induction of hepatic fatty acid oxidation via HNF-4α and PGC-1α dependent mechanism. Our findings help to explain why methyl-donor supplementation halts the progression of NAFLD in obese subjects. PRMT1 inhibitors are being developed to treat various cancers 67-69, it is necessary to evaluate the potential side effects of PRMT1 inhibitor treatments on hepatic lipid metabolism and liver damages.

## Supplementary Material

Supplementary figures and table.

## Figures and Tables

**Table 1:** List of sequences of shRNAs

Name	Sequence	Vector	Ref.
shPRMT1-1	5'-TGAGGACATGACATCCAAATTCAAGAGATTTGGATGTCATGTCCTCAGCTTTTTT-3'	pAV-U6-GFP	Vigene, 31
shPRMT1-2	5'-AACTCCATGTTTCACAATCTTCAAGAGAGATTGTGAAACATGGAGTTGCTTTTTT-3'	pAV-U6-GFP	Vigene
shPRMT1-3	5'-ATTAAAGACGTGGCCATCATTCAAGAGA TGATGGCCACGTCTTTAATGCTTTTTT-3'	pAV-U6-GFP	Vigene
shPGC-1α-1	5'-GGTGGATTGAAGTGGTGTAGACTCGAGT CTACACCACTTCAA TCCACCTTTTTT-3'	pLKD-U6-MSC-CMV-Puro	40
shPGC-1α-2	5'-CTGACTTCGAGCTGTACTTCTCAAGAGA AAGTACAGCTCGAA GTCAGTTTTTT-3'	pLKD-U6-MSC-CMV-Puro	43
shHNF-4α-1	5'-GCGAACTCCTTCTGGATGATTCAAGAGA TCATCCAGAAGGAG TTCGCTTTTTT-3'	pLKD-U6-MSC-CMV-Puro	42
shHNF-4α-2	5'-GGTGCCAACCTCAATTCATCCCTCGAGG GATGAATTGAGGTT GGCACCTTTTTT-3'	pLKD-U6-MSC-CMV-Puro	41

**Table 2:** List of primary antibodies

Antibody name	Catalog number	Application	Producer
Rabbit anti-PRMT1	#07-404	WB, IHC	Millipore
Rabbit anti-β-actin	#8457	WB	CST
Rabbit anti-PGC-1α	ab54481	WB, IHC	Abcam
Rabbit anti-HNF-4α	ab181604	WB, ChIP	Abcam
Rabbit anti-CD11b	ab133357	WB	Abcam
Rabbit anti-albumin	ab207327	WB, IHC	Abcam

**Table 3:** Sequences of primers used for qPCR or plasmids constructs in this study.

Species	Gene Name	Primer sequences (5'-3')	
		Forward	Reverse
Mouse	PRMT1	TGGAAGCAGACTGTGTTCTAC	ACACAGCTGACCCTTGAAG
	Albumin	ACAAGGACACCTGCTTCTC	AGTCCTGAGTCTTCATGTCTTT
	F4/80	CTTTGGCTATGGGCTTCCAGTC	GCAAGGAGGACAGAGTTTATCGTG
	α-SMA	AAACAGGAATACGACGAAG	CAGGAATGATTTGGAAAGGA
	ACC-1	ACAGTGGAGCTAGAATTGGAC	ACTTCCCGACCAAGGACTTTG
	SCD1	TGCTATCGGGGTGTTAATGA	TCTTGTGGCATGGTTAATCCTA
	PPARγ	TCGCTGATGCACTGCCTATG	GAGAGGTCCACAGAGCTGATT
	SREBP-1c	TGACCCGGCTATTCCGTGA	CTGGGCTGAGCAATACAGTTC
	TGH	AGCTTTGTATCGGCCATGAG	CTGAGTTGAGGCACCAATCTT
	MGAT1	CGGAAGCTGATCTACACTGTT	AGCTCCTCTAGGTATGTCTGAT
	Cideb	AGCCTCAACGTGAAAGCTAC	TTGCAGCAGCGAGGAAG
	CPT1α	CAGAGGATGGACACTGTAAAGG	CGGCACTTCTTGATCAAGCC
	ACOX1	TAACTTCCTCACTCGAAGCCA	AGTTCCATGACCCATCTCTGTC
	Ehhadh	ATGGCTGAGTATCTGAGGCTG	GGTCCAAACTAGCTTTCTGGAG
	Acaa1b	CAGGACGTGAAGCTAAAGCCT	CTCCGAAGTTATCCCCATAGGAA
	SCAD	ACCAAAGCTTGGATCACCAACTCC	AACCAGGAAGGCACTGATACCCTT
	LCAD	CTTGCTTGGCATCAACATCGCAGA	ATTGGAGTACGCTTGCTCTTCCCA
	VLCAD	GGCCAAGCTGGTGAAACACAAGAA	ACAGAACCACCACCATGGCATAGA
	PGC-1α	AAGTGGTGTAGCGACCAATCG	AATGAGGGCAATCCGTCTTCA
	HNF-4α	CTAACACGATGCCCTCTCAC	GCAGGAGCTTGTAGGATTCAG
	β-actin	ACCTTCTACAATGAGCTGCG	CTGGATGGCTACGTACATGG
	PGC-1α_Promoter_	AGTGACAGCCCAGCCTACT	GACACACAGCACACACTCATGC
Human	PRMT1	CTTTGACTCCTACGCACACTT	GTGCCGGTTATGAAACATGGA
	PGC-1α	TGAAGACGGATTGCCCTCATT	GCTGGTGCCAGTAAGAGCTT
	HNF-4α	CGAAGGTCAAGCTATGAGGACA	ATCTGCGATGCTGGCAATCT
	β-actin	GTCTTCCCCTCCATCGTG	GTACTTCAGGGTGAGGATGC

## Abbreviations

AAV: adeno-associated virus
Acaa1b: acetyl-coenzyme A acyltransferase 1b
ACC: acetyl-CoA carboxylase
ACOX1: acyl‐CoA oxidase 1
ALT: alanine aminotransferase
ASMs: acid-soluble metabolites
AST: aspartate aminotransferase
AUC: area under the curve
BMI: body mass index
Cideb: cell death-inducing DFF45-like effector b
ChIP: chromatin immunoprecipitation
CPT1α: carnitine-dependent transport-1α
DBP: diastolic blood pressure
DNA: deoxyribonucleic acid
GFP: green fluorescent protein
Ehhadh: enoyl-CoA hydratase and 3-hydroxyaryl CoA dehydrogenase
FAO: fatty acid oxidation
FoxO1: forkhead box protein O1
gDNA: genomic DNA
GTT: intra-peritoneal glucose tolerance test
HDL: high-density lipoprotein
HE: hematoxylin and eosin
H4R3: histone 4 Arginine 3
H4R3me2a: H4R3 dimethyl Asymmetric
HFD: high-fat diet
HNF-4α: hepatocyte nuclear factor-4α
HOMA-IR: homeostasis model assessment of insulin resistance
HRP: Horseradish peroxidase
IHC: immuno-histochemistry
IHCs: intrahepatic parenchymal cells
IP: intraperitoneal
ITT: insulin tolerance test
LCAD: long-chain acyl-CoA dehydrogenases
LDL: low-density lipoprotein
MGAT1: monoacylglycerol acyltransferase 1
mRNA: messenger ribonucleic acid
NAFLD: non-alcoholic fatty liver disease
NASH: non-alcoholic steatohepatitis
PEI: polyethylenimine
PGC-1α: peroxisome proliferator-activated receptor gamma coactivator 1-α
PPARγ: peroxisome proliferator-activated receptor γ
PRMT1: protein arginine methyltransferases 1
PVDF: polyvinylidene fluoride
QPCR: quantitative real-time polymerase chain reaction
RIPA: radio-immunoprecipitation assay
SAM: s-adenosylmethionine
SCAD: short-chain acyl-CoA dehydrogenases
SCD1: stearoyl CoA desaturase 1
SDS-PAGE: sodium dodecyl sulphate polyacrylamide gel electrophoresis
SEM: standard error of mean
SREBP-1c: sterol regulatory element-binding protein 1
STC: standard chow diet
TC: total cholesterol
TG: triglyceride
TGH: triacylglycerol hydrolase
VLCAD: very-long-chain acyl-CoA dehydrogenases
VLDL: very-low-density lipoprotein
WT: wildtype
