# Supplementary material for: Hepatic PRMT1 ameliorates diet-induced hepatic steatosis via induction of PGC1α
Source: Theranostics. 2022 Feb 28;12(6):2502–18. doi: 10.7150/thno.63824 (PMC8965489; doi:10.7150/thno.63824)
Supplement: Supplementary file 1 — Supplementary figures and table. [file thnov12p2502s1.pdf]

Hepatic PRMT1 ameliorates diet-induced hepatic steatosis via induction of PGC1α

Lu XU, Zhe HUANG, Tak-ho LO, Jimmy Tsz Hang LEE, Ranyao YANG, Xingqun YAN, Dewei YE, Aimin XU, Chi-Ming WONG.

Table of contents

Supplementary Fig. 1……………………………………………………………….2

Supplementary Fig. 2..……………………………………………………..………….3

Supplementary Fig. 3..……………………………………………………..………….4

Supplementary Fig. 4..……………………………………………………..………….5

Supplementary Fig. 5..……………………………………………………..………….6

Supplementary Fig. 6..……………………………………………………..………….7

Supplementary Fig. 7..……………………………………………………..………….8

Supplementary Table 1….………………………………………………………….9

Supplementary Figure 1

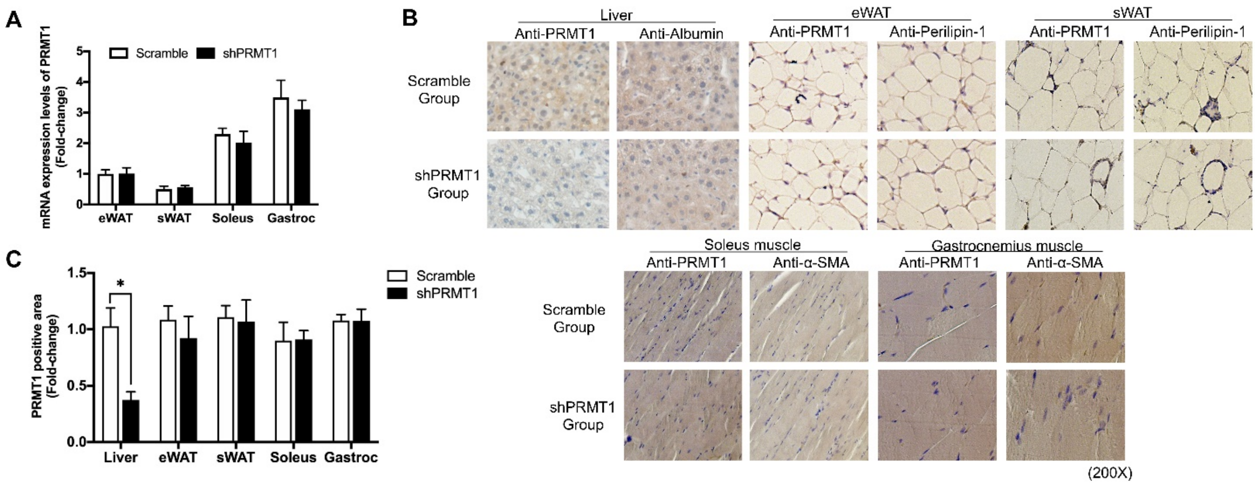

Supplementary Fig. 1. Related to Fig. 2. Specificity of PRMT1 knock-down. Eight-week-old male C57BL/6N mice were infected with 3×10^{11} copies of AAV encoding U6-PRMT1 shRNA (shPRMT1) or scramble control (Scramble) shRNA for 9 weeks upon STC feeding. **A.** mRNA expression levels of PRMT1 in epididymal white adipose tissue (eWAT), subcutaneous WAT (sWAT), soleus muscles and gastrocnemius muscles (Gastroc) as determined by real-time PCR analysis. mRNA expression levels of PRMT1 among different tissues were normalized to the expression of mouse β-actin. mRNA expression levels of PRMT1 in eWAT with Scramble shRNA infected were set as 1 for fold-change calculation. There was no significant difference in the mRNA expression levels of PRMT1 between Scramble and shPRMT1 infected groups. **B.** Representative images of immunohistochemical staining of PRMT1 in various tissues. Liver specific marker Albumin for liver sections, perilipin-1 for adipose tissues and α-smooth muscle actin (α-SMA) for soleus muscles and gastrocnemius muscles sections. (200X) **C.** Quantification of PRMT1 expression level by ImageJ. Scramble groups were set as 1 for fold-change calculation. Data represent as mean ± SEM; n = 5-8 per group; repeated with three independent experiments.

Supplementary Figure 2

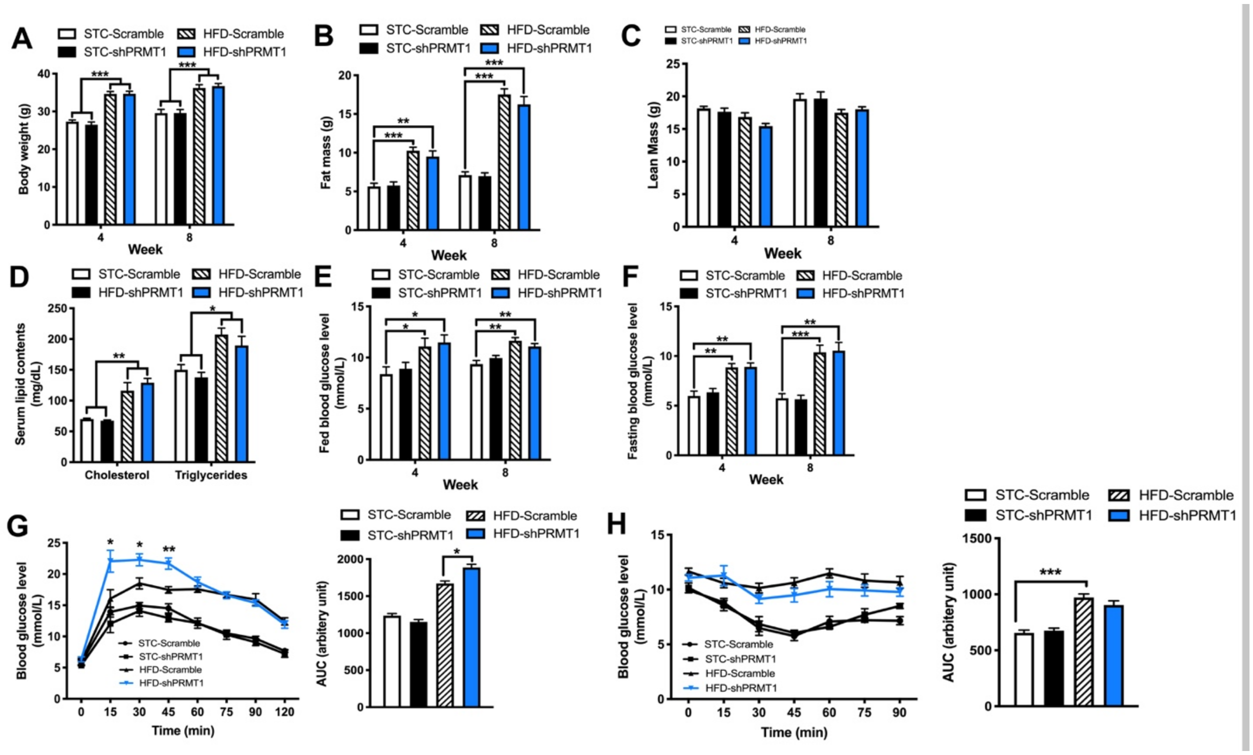

Supplementary Fig. 2. Related to Fig. 2. Knockdown of PRMT1 exacerbates diet-induced insulin resistance. Eight-week-old male C57BL/6N mice were infected with 3 × 10^{11} copies of AAV encoding U6-PRMT1 shRNA (shPRMT1) or scramble control (Scramble) shRNA for 9 weeks upon either STC or HFD feeding, respectively. Body weight (**A**), fat mass (**B**) and lean mass (**C**) at different weeks since HFD feeding were measured. **D**. Serum lipid contents at week of 10 were tested. Fed (**E**) and fasting (**F**) blood glucose levels of mice at different weeks since HFD feeding were measured. **G**. Glucose tolerance test (GTT, 1 g/kg, left) and area under curve (AUC, right) of serum glucose at week of 9. **H**. Insulin tolerance test (ITT, 0.5 U/kg, left) and AUC (right) of serum glucose at week of 9. Data represent as mean ± SEM; n = 5-8 per group; repeated with three independent experiments; **P* < 0.05, ***P* < 0.01 and ****P* < 0.001 were compared as indicated, otherwise were compared between HFD-Scrambled group and HFD-shPRMT1 group.

Supplementary Figure 3

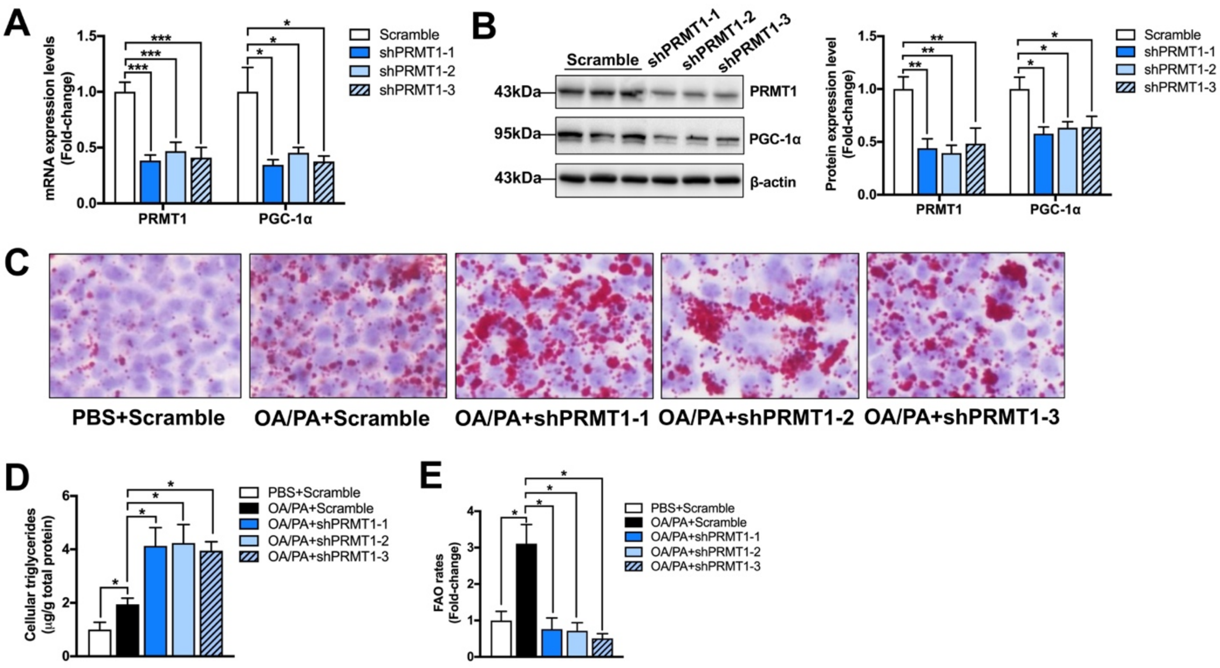

Supplementary Fig. 3. Related to Fig. 3 Knockdown of PRMT1 exaggerates oleic acid (OA) and palmitic acid (PA)-induced lipid deposition on Hepa1-6 cells via the down-regulation of PGC-1α. Hepa1-6 was transfected with either shScramble (Scramble) or shPRMT1-1 or shPRMT1-2 or shPRMT1-3 plasmids for 72 hours and then incubated with PBS or 0.8 mM OA/PA (ratio 2:1) for another 24 hours. **A**. mRNA expression levels of PRMT1 and PGC-1α as determined by qPCR analysis. **B**. Protein expression of PRMT1 and PGC-1α as determined by Western blotting (left). Quantification of protein expression levels of PRMT1 (middle) and PGC-1α (right). **C**. Representative images of Oil Red O staining of Hepa1-6 cells. (200X) **D**. Cellular triglycerides levels were normalized by total protein contents of cell lysates used for lipid extraction. **E**. Cellular fatty acid oxidation (FAO) rates were determined by FAO Kit (BMR service, State University of New York at Buffalo). mRNA expression levels of the target genes were normalized to the expression of mouse β-actin. Control group was set as 1 for fold-change calculation. Data represent mean ± SEM; n = 4 per group; **P* < 0.05, ***P* < 0.01, ****P* < 0.001.

Supplementary Figure 4

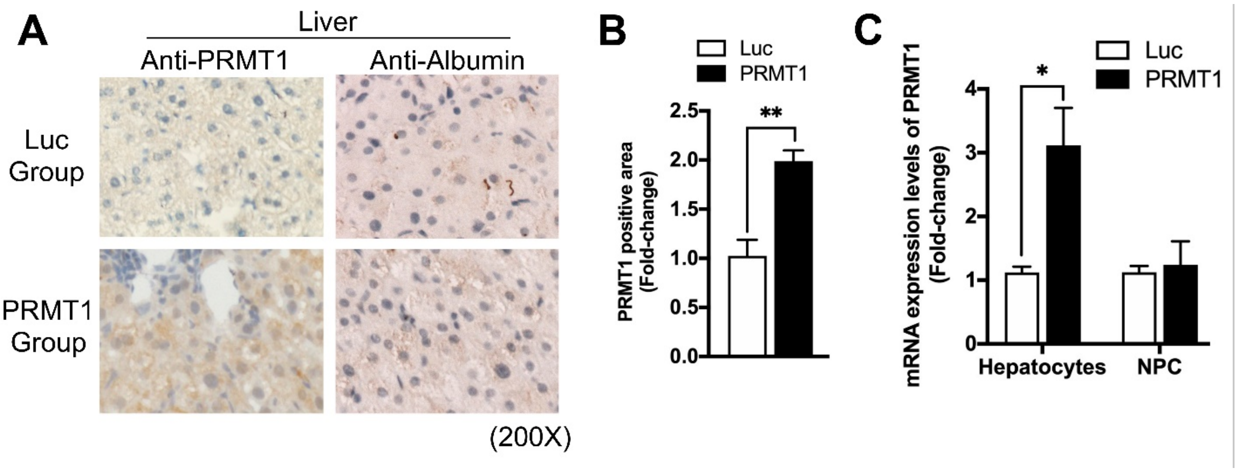

Supplementary Fig. 4. Related to Fig. 4. Specificity of overexpressing PRMT1. Eight-week-old male C57BL/6N mice were infected with 3×10^{11} copies of wild-type PRMT1 (PRMT1 WT) or Luciferase (Luc), respectively, for 12 weeks upon HFD feeding. **A.** Representative images of immunohistochemical staining of PRMT1 and liver specific marker Albumin for liver sections. (200X). **B.** Quantification of PRMT1 expression level by ImageJ. **C.** qPCR analysis of PRMT1 in hepatocytes or non-parenchymal cell (NPC) fraction from liver of AAV-Luc and AAV-PRMT1 mice fed with HFD. mRNA expression levels of the target genes were normalized to the expression of β-actin. Luciferase group was set as 1 for fold-change calculation. Data represent as mean ± SEM; n = 5-8 per group; repeated with three independent experiments. *$P < 0.05$, **$P < 0.01$.

Supplementary Figure 5

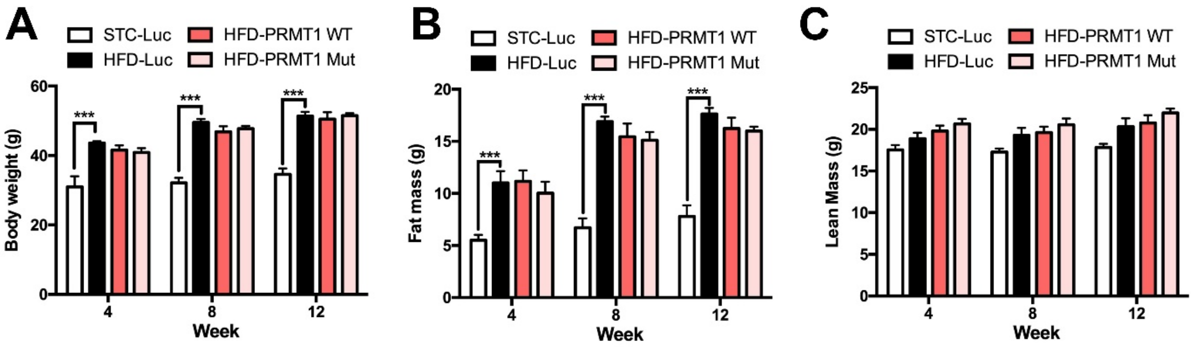

Supplementary Fig. 5. Related to Fig. 4. Liver-specific over-expression of PRMT1 methyltransferase inactive mutant PRMT1^{G80R} has no protective effect on insulin sensitivity in mice upon long-term HFD feeding. Eight-week-old male C57BL/6N mice were infected with 3 × 10^{11} copies of AAV encoding wild-type PRMT1 (PRMT1 WT), methyltransferase inactive mutant PRMT1^{G80R} (PRMT1 Mut) or Luciferase (Luc) for 12 weeks upon HFD feeding, respectively. Body weight (**A)**, fat mass (**B)** and lean mass (**C)** at week of 10 were tested. Data represent as mean ± SEM; n = 5-8 per group; repeated with three independent experiments; ****P* < 0.001 were compared with STC-Luc group.

Supplementary Figure 6

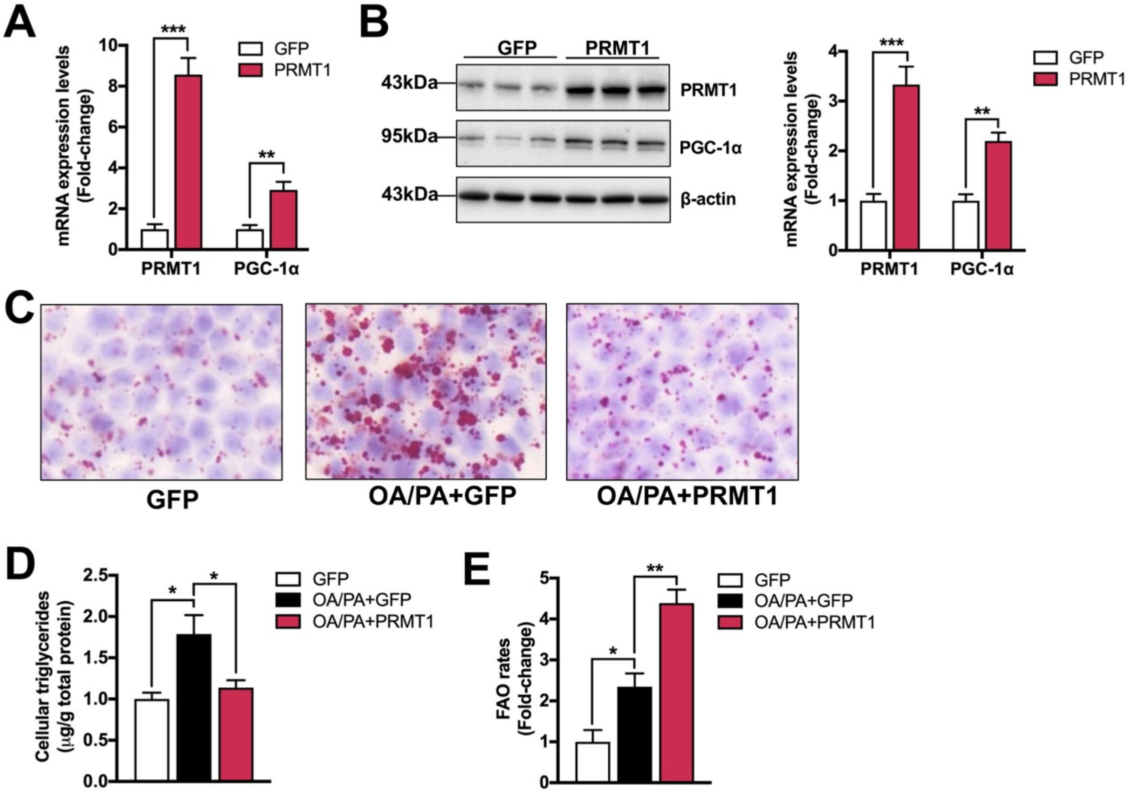

Supplementary Fig. 6. Related to Fig. 5. Over-expressing PRMT1 decreases oleic acid (OA) and palmitic acid (PA)-induced lipid accumulation and increases fatty acid oxidation on Hepa1-6 cells. Hepa1-6 was transfected with either GFP or PRMT1 over-expressing plasmids for 48 hours and then incubated with PBS or 0.8 mM OA/PA (ratio 2:1) for another 24 hours. **A.** mRNA expression levels of PRMT1 and PGC-1α as determined by qPCR analysis. **B**. Protein expression of PRMT1 and PGC-1α as determined by Western blotting (left). Quantification of protein expression levels of PRMT1 (middle) and PGC-1α (right). **C**. Representative images of Oil Red O staining of Hepa1-6 cells. (200X) **D.** Cellular triglycerides levels were normalized by total protein contents of cell lysates used for lipid extraction. **E.** Cellular fatty acid oxidation (FAO) rates were determined by a FAO Kit. mRNA expression levels of the target genes were normalized to the expression of mouse β-actin. Control group was set as 1 for fold-change calculation. Data represent as mean ± SEM; n = 4 per group; repeated with three independent experiments; **P* < 0.05, ***P* < 0.01, ****P* < 0.001.

Supplementary Figure 7

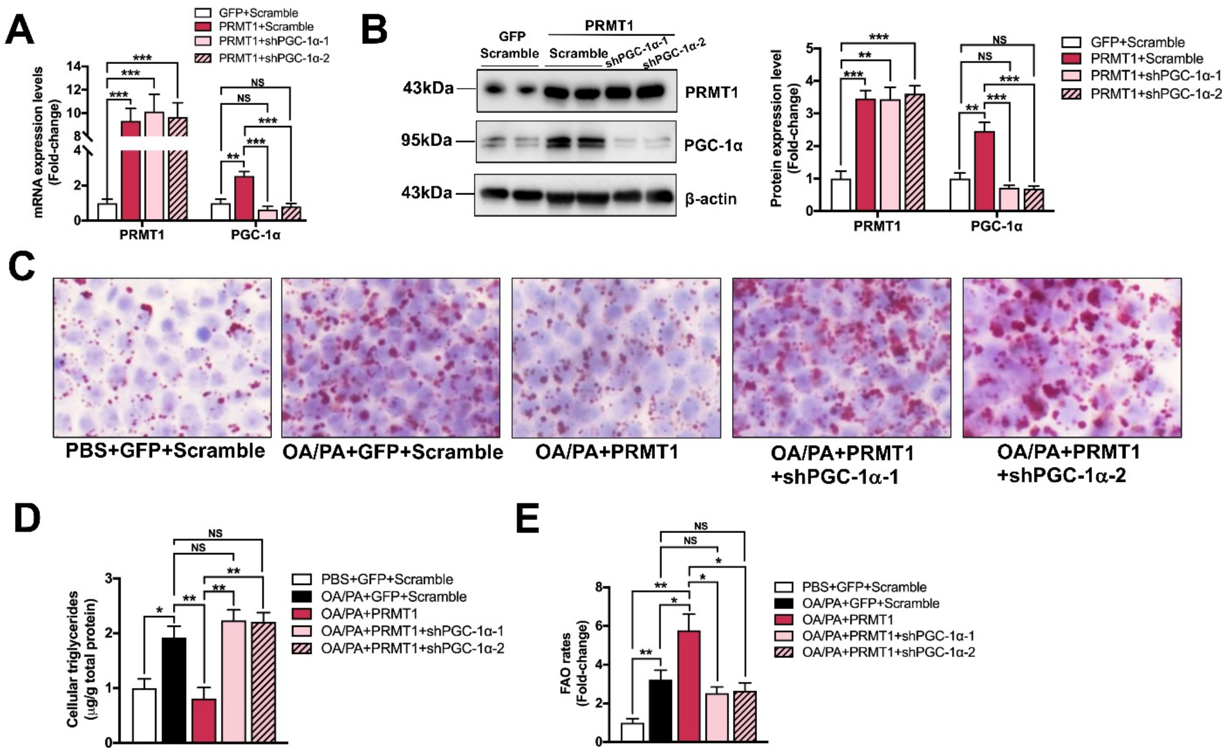

Supplementary Fig. 7. Related to Fig. 5. PGC-1α is required for PRMT1-mediated alleviation of oleic acid (OA) and palmitic acid (PA)-induced lipid deposition on Hepa1-6 cells. Hepa1-6 was transfected with either GFP or PRMT1 over-expressing plasmids for 24 hours and then transfected with shScramble (Scramble) or shPGC-1α-1 or shPGC-1α-2 plasmids for another 72 hours. Cells were then incubated with PBS or 0.8 mM OA/PA (ratio 2:1) for another 24 hours. **A.** mRNA expression levels of PRMT1 and PGC-1α as determined by qPCR analysis. **B**. Protein expression of PRMT1 and PGC-1α as determined by Western blotting (left). Quantification of protein expression levels of PRMT1 (middle) and PGC-1α (right). **C**. Representative images of Oil Red O staining of Hepa1-6 cells. (200X) **D.** Cellular triglycerides levels were normalized by total protein contents of cell lysates used for lipid extraction. **E.** Cellular fatty acid oxidation (FAO) rates were determined by a FAO Kit. Quantification of protein expression levels of PRMT1 and PGC-1α (from left to right). mRNA expression levels of the target genes were normalized to the expression of mouse β-actin. Control group was set as 1 for fold-change calculation unless mentioned otherwise. Data represent as mean ± SEM; n = 4 per group; repeated with three independent experiments; *P < 0.05, **P < 0.01, ***P < 0.001, NS, not significant.

Supplementary Table 1. Baseline characteristics of study cohorts.

	**Non-steatosis (n = 4)**	**Steatosis (n = 8)**
**Male / Female**	4/0	3/5
**BMI (kg/m^2)**	33.6 ± 5.3	39.0 ± 7.5
**Age (years)**	24.3 ± 3.3	28.4 ± 5.9
**Fasting Glucose (mmol/L)**	5.2 ± 0.4	8.0 ± 5.8
**Fasting Insulin (mU/L)**	17.3 ± 3.2	18.8 ± 11.3
**HOMA-IR**	4.0 ± 1.0	5.9 ± 4.0
**SBP (mmHg)**	126.5 ± 5.0	130.0 ± 14.3
**DPB (mmHg)**	85.3 ± 3.5	83.1 ± 17.0
**Triglycerides (mmol/L)**	1.4 ± 0.8	6.3 ± 13.9
**Total Cholesterol (mmol/L)**	5.5 ± 0.5	5.2 ± 1.6
**HDL (mmol/L)**	1.1 ± 0.1	1.1 ± 0.5
**LDL (mmol/L)**	3.5 ± 0.4	2.7 ± 1.1
**ALT (U/L)**	15.3 ± 6.7	92.9 ± 73.5
**AST (U/L)**	17.5 ± 1.3	48.0 ± 30.6
**γGGT (U/L)**	22.3 ± 4.6	19.4 ± 3.8

Abbreviations: BMI, body mass index; HOMA-IR, homeostasis model assessment of insulin resistance; SBP, systolic blood pressure; DBP, diastolic blood pressure; HDL, high density lipoproteins; LDL, low density lipoproteins; ALT, alanine aminotransferase; AST, aspartate transaminase; γGGT, γ-glutamyl transpeptidase.
There was no significant difference between Non-steatosis and Steatosis groups in these parameters.
